# Supplementary material for: Cancer Loyalty Card Study (CLOCS): protocol for an observational case–control study focusing on the patient interval in ovarian cancer diagnosis
Source: BMJ Open. 2020 Sep 8;10(9):e037459. doi: 10.1136/bmjopen-2020-037459 (PMC7484869; doi:10.1136/bmjopen-2020-037459)
Supplement: Author's manuscript [file bmjopen-2020-037459.draft_revisions.pdf]

BMJ Open is committed to open peer review. As part of this commitment we make the peer review history of every article we publish publicly available.

When an article is published we post the peer reviewers' comments and the authors' responses online. We also post the versions of the paper that were used during peer review. These are the versions that the peer review comments apply to.

The versions of the paper that follow are the versions that were submitted during the peer review process. They are not the versions of record or the final published versions. They should not be cited or distributed as the published version of this manuscript.

BMJ Open is an open access journal and the full, final, typeset and author-corrected version of record of the manuscript is available on our site with no access controls, subscription charges or pay-per-view fees (http://bmjopen.bmj.com).

If you have any questions on BMJ Open's open peer review process please email
info.bmjopen@bmj.com

BMJ Open

Cancer Loyalty Card Study (CLOCS): protocol for an observational case-control study focusing on the patient interval in ovarian cancer diagnosis

Journal:	*BMJ Open*
Manuscript ID	bmjopen-2020-037459
Article Type:	Protocol
Date Submitted by the Author:	03-Feb-2020

Complete List of Authors:	Brewer, Hannah; Imperial College London, Surgery and Cancer<br>Hirst, Yasemin; University College London, Epidemiology and Public Health<br>Sundar, Sudha; University of Birmingham, Cancer Sciences<br>Chadeau-Hyam, Marc; Imperial College London, MRC Centre for Environment and Health; Imperial College London, Epidemiology and Biostatistics, School of Public Health<br>Flanagan, James; Imperial College London, Surgery and Cancer

Keywords:	ONCOLOGY, Gynaecological oncology < GYNAECOLOGY, Epidemiology < ONCOLOGY, PUBLIC HEALTH

SCHOLARONE™
Manuscripts

I, the Submitting Author has the right to grant and does grant on behalf of all authors of the Work (as defined in the below author licence), an exclusive licence and/or a non-exclusive licence for contributions from authors who are: i) UK Crown employees; ii) where BMJ has agreed a CC-BY licence shall apply, and/or iii) in accordance with the terms applicable for US Federal Government officers or employees acting as part of their official duties; on a worldwide, perpetual, irrevocable, royalty-free basis to BMJ Publishing Group Ltd ("BMJ") its licensees and where the relevant Journal is co-owned by BMJ to the co-owners of the Journal, to publish the Work in this journal and any other BMJ products and to exploit all rights, as set out in our licence.

The Submitting Author accepts and understands that any supply made under these terms is made by BMJ to the Submitting Author unless you are acting as an employee on behalf of your employer or a postgraduate student of an affiliated institution which is paying any applicable article publishing charge ("APC") for Open Access articles. Where the Submitting Author wishes to make the Work available on an Open Access basis (and intends to pay the relevant APC), the terms of reuse of such Open Access shall be governed by a Creative Commons licence – details of these licences and which Creative Commons licence will apply to this Work are set out in our licence referred to above.

Other than as permitted in any relevant BMJ Author's Self Archiving Policies, I confirm this Work has not been accepted for publication elsewhere, is not being considered for publication elsewhere and does not duplicate material already published. I confirm all authors consent to publication of this Work and authorise the granting of this licence.

For peer review only

TITLE

Cancer Loyalty Card Study (CLOCS): protocol for an observational case-control study focusing on the patient interval in ovarian cancer diagnosis

Short Title

Cancer Loyalty Card Study (CLOCS): protocol for a case-control study

AUTHORS

Hannah R. Brewer[1], Yasemin Hirst[2], Sudha Sundar[3,4], Marc Chadeau-Hyam[5,6] and James M. Flanagan[1]

1. Department of Surgery and Cancer, Imperial College London, London, UK
2. Department of Behavioural Science and Health, University College London, London, UK
3. Pan-Birmingham Gynaecological Cancer Centre, City Hospital, Birmingham, UK.
4. Institute of Cancer and Genomic Sciences, University of Birmingham, Birmingham, UK.
5. MRC Centre for Environment and Health, Imperial College London, London, United Kingdom.
6. Department of Epidemiology and Biostatistics, School of Public Health, Imperial College London, London, United Kingdom.

Address for Correspondence:

Dr. James M. Flanagan

Division of Cancer, Department of Surgery and Cancer,

Faculty of Medicine, Imperial College London

4th Floor IRDB, Hammersmith Campus, Du Cane Road, London W12 0NN

Tel: 020 75941804; email: j.flanagan@imperial.ac.uk

For peer review only - http://bmjopen.bmj.com/site/about/guidelines.xhtml

For peer review only

ABSTRACT

Introduction

Ovarian Cancer is the eighth most common cancer in women worldwide, and about one in five women with ovarian cancer do not receive treatment, because they are too unwell by the time they are diagnosed. Symptoms of ovarian cancer are non-specific or can be associated with other common conditions, and women experiencing these symptoms have been shown to self-manage them using over-the-counter medication. Results from a recent proof of concept study suggest there may be an increase in the purchases of pain killers and indigestion medication 10-12 months before ovarian cancer diagnosis. We propose a case-control study, as part of a larger project called the Cancer Loyalty Card Study (CLOCS), to investigate whether a significant change in medication purchases could be an indication for early signs of ovarian cancer, using data already collected through store loyalty cards.

Methods and Analysis

Using a retrospective case-control design, we aim to recruit 500 women diagnosed with ovarian cancer (cases) and 500 women without ovarian cancer (controls) in the United Kingdom who hold a loyalty card with at least one participating high street retailer. We will use pre-existing loyalty card data to compare past purchase patterns of cases with those of controls. In order to assess ovarian cancer risk in participants and their purchase patterns, we will collect information from participants on ovarian cancer risk factors and clinical data including symptoms experienced before diagnosis from recruited women with ovarian cancer.

Ethics and dissemination

CLOCS was reviewed and approved by the North West - Greater Manchester South Research Ethics Committee (19/NW/0427). Outcomes of the study will be disseminated through academic publications, the study website, social media, and a report to the research sites that support the study once results are published.

Study Registration Numbers

This study is registered with the ISRCTN (14897082), is an NIHR portfolio study (CPMS 43323), and is registered on clinicaltrials.gov (NCT03994653).

Keywords:

Ovarian cancer, early detection, purchase behaviour, observational case-control study, commercial data

For peer review only - http://bmjopen.bmj.com/site/about/guidelines.xhtml

Article Summary

Strengths and limitations of this study

- This study is a novel approach to investigate whether routinely collected commercial purchase data can be used to reduce delays in ovarian cancer diagnosis.
- Ovarian cancer advocates have been involved in the conception, design and conduct of the study. A key strength of the study is the detailed information available for purchases recorded for up to 7 years prior to enrolment.
- A key limitation is that loyalty card holders may not use their cards for every purchase, may shop at other stores not being accessed in this study and may be purchasing items for other family members.
- In general, due to the retrospective nature of case-control studies, there is potential for the misreporting of exposures in the risk factor questionnaire by cases and controls (recall bias).

INTRODUCTION

Approximately 7,200 new cases of ovarian cancer are diagnosed each year in the UK, and with over 4,200 women dying from the disease each year, it is a particularly lethal form of cancer[1]. Diagnosing ovarian cancer in primary and secondary care settings among symptomatic patients is a challenge due to their low positive predictive value of symptoms and symptom presentation at various stages of the ovarian cancer prognosis[2,3]. Symptoms, such as increased abdominal size, pelvic pain, abdominal pain and bloating, feeling full quickly and difficulty eating have been identified as high risk symptoms but equally it has been reported that the absence of a symptom does not help ruling out ovarian cancer[4,5]. For patients whose symptoms do not warrant an immediate referral earlier presentation in primary care is crucial to reduce delays in diagnosis and subsequent treatment.

However, symptom recognition and help-seeking in primary care are also not without challenges. While in some cases patients may present very quickly, for some patients it could take up to 12 months before they seek any medical help. It has been shown that greater awareness of symptoms alone does not facilitate help seeking because symptoms are often perceived as vague or trivial[6,7]. The major reasons for not presenting to the primary care

For peer review only - http://bmjopen.bmj.com/site/about/guidelines.xhtml

are “not wanting to waste the GP’s time”[8], normalisation of these symptoms[9], and few women believing that their symptom(s) might be a sign of cancer[10]. Women might choose to self-manage such symptoms by seeking advice from sources other than a primary care physician, e.g. pharmacists, family, internet, and using over-the-counter medication[11]. There are currently no organised screening programmes offered to women that could help detect ovarian cancer at an earlier stage, and there is little evidence for effective interventions aiming to improve symptom recognition and help-seeking.

Therefore, novel methods are needed to improve earlier detection of low-volume disease or earlier stage to improve survival[12]. A recent proof of concept study investigated whether purchase information collected through the high street retailers’ loyalty cards (e.g. store points cards) could be used to identify women’s self-care behaviours prior to presenting to health care professionals. This study included data from a major high street retailer’s loyalty card of women who have previously been diagnosed with ovarian cancer and consented their past purchase data to be provided to the researchers. The study suggested that there may be an increase in the purchases of pain killers and indigestion tables up to 1 0-12 months before diagnosis[13]. This proof-of-concept study provided a new model of investigation for symptom recognition and help-seeking among ovarian cancer patients. It highlighted potential strengths and limitations in its methodology for future research. Building on these results, self-care behaviours of ovarian cancer patients will be further investigated with a case-control study within a larger project called Cancer Loyalty Card Study (CLOCS). CLOCS hypothesises that a significant change in purchasing behaviour, primarily an increase in self-medication or self-care behaviours to treat symptoms prior to presentation in primary care (see Figure 1), could be an indication for early signs of ovarian cancer.

Study aims

The primary aim of this case-control study is to investigate pre-diagnosis purchasing behaviours of ovarian cancer patients (cases) with women without a previous ovarian cancer diagnosis (controls) using data collected through two major high street retailers’ loyalty card schemes.

The secondary aim of CLOCS will be to define a purchase threshold as an “alert” about ovarian cancer symptoms in individuals and determining the predictive utility of purchasing behaviours in the early detection of ovarian cancer based on ovarian cancer risk.

For peer review only - http://bmjopen.bmj.com/site/about/guidelines.xhtml

METHODS AND ANALYSIS

Study design and setting

CLOCS will use a retrospective case-control design to compare past purchase patterns of women with and without ovarian cancer using already existing data gathered by two major high street retailers. Furthermore, we will collect data on risk factors for ovarian cancer, symptoms experienced before diagnosis and clinical data from ovarian cancer patients using self-reported questionnaires.

CLOCS will be recruiting across the United Kingdom from at least 40 National Health Service (NHS) Trusts and Hospitals and recruit healthy volunteers using online recruitment.

Study timeline

The CLOCS case-control study will take place from 1st November 2019 until 1st February 2022.

Participants

Inclusion/Exclusion Criteria

Cases

Women, at least 18 years old, diagnosed with ovarian cancer (all subtypes will be included) within the previous 2 years from the recruitment date who own at least one participating high street retailer loyalty card are eligible to join CLOCS case-control study as cases.

There are no further clinical criteria for patients to be excluded from this study. Patients' usual care will continue to be provided by the health care professionals, and CLOCS participation does not exclude patients from participating in other clinical or non-clinical studies.

Controls

Women, at least 18 years old, who have not been diagnosed with ovarian cancer and own at least one of the loyalty cards from the participating retailers are eligible to join the CLOCS as controls. We are not excluding women with previous cancer diagnosis, or women who may have had their ovaries removed for reasons other than cancer; however, these will be considered when cases are matched with controls.

Recruitment

The CLOCS case-control study recruitment process is outlined in figure 2 and described further detail below.

For peer review only - http://bmjopen.bmj.com/site/about/guidelines.xhtml

Cases

Eligible ovarian cancer patients will be recruited by members of their healthcare teams at approximately 40 NHS Trusts and Hospitals across the United Kingdom that have agreed to approach patients at clinics. These healthcare teams are registered as research sites and will have a principal investigator (PI) leading the recruitment to CLOCS. The PI and the research nurses will facilitate recruitment by identifying patients when they think it is suitable to approach patients based on patients' overall well-being at a suitable time (e.g. at waiting areas, during chemotherapy sessions), then will provide patients with the study information to consider participation. Patients will be given a paper-based participant pack that includes study consent form including consents for providing access to data collected by the retailers, self-report questionnaire, and a clinical questionnaire to be completed by the clinical staff facilitating the recruitment. If they wish to participate, patients can complete all the study documents at the recruitment site and post it back to the research team using the free-post envelope provided in the study pack, or they are also given the option to consider participation at home provided that the clinical questionnaire has been completed by a member their clinical team. The research sites will not need to record any data from the participants. The data collection from the cases will be a one-off process and no further contact with the patient is required for the study.

Controls

The control participants to the study will be recruited through the study website (www.clocsproject.org.uk) where data collection will be safeguarded using an encrypted web form that includes study consent and the self-report questionnaire. The website has been developed and maintained by NDP limited. The questionnaire for women without ovarian cancer will be confined to risk factors. The online participation will take up to 15 minutes and data will be transferred immediately to the secure CLOCS server (see figure 3). The website approach was adopted as one of the outcomes of the proof-of-concept study. It aims to provide a transparent environment where potential participants can make informed decisions about participation in CLOCS, and the data risks are minimised using encryption. We have carefully designed the website to inform the general public about how we will process their data in CLOCS, how they can withdraw from the study if they wish to do so and use it as a platform to provide feedback as we progress in the larger CLOCS project. The research team will use several methods to recruit control participants to the study which will include a press release carried out by the project funders and the Imperial College London public engagement team, dissemination of study flyers and leaflets, and social media

For peer review only - http://bmjopen.bmj.com/site/about/guidelines.xhtml

marketing techniques on Twitter and Facebook using paid advertisement and unpaid methods. The application of the social media recruitment methods will allow researchers to reach a larger cohort in the UK. The application of both paper-based and online recruitment methods will be carried out throughout CLOCS and will be recorded. The efficacy of these methods will be reported in peer-reviewed journals to inform online research recruitment to future case-control studies.

Data sources and variables included

Loyalty Card Data (For cases and controls)

Once participants consent for the CLOCS team to request their loyalty card data from the retailers on their behalf, the study will include individuals' past purchase data from up to seven years prior to the date of study consent. The purchase data will include each individual purchase, date of purchase, the location (i.e. store postcode), and the product categorisation that is provided by the retailers. There will be no additional individual information recorded by the study team from this dataset and no additional individual information will be requested from the retailers. Please note that at this stage of the study, we will not be naming the high street retailers in the publications and in our direct communications, but they are named to the potential participants when they agree to take part.

Ovarian Cancer Risk Questionnaire (For Cases and Controls)

In order to stratify the purchase differences between cases and controls based on ovarian cancer risk, we designed a questionnaire on ovarian cancer risk factors that will allow us to determine individuals' ovarian cancer risk. Both cases and control participants will be asked to complete a short questionnaire about ethnicity, marital status, and the following well-established ovarian cancer risk factors: body mass index, age at menarche, menopausal status, age at menopause, parity, breastfeeding, hysterectomy, tubal ligation, cancer history, endometriosis, aspirin use, oral contraceptive use, hormone replacement therapy (HRT), family history of ovarian and breast cancers, vaping, and cigarette smoking[14]. These factors will be used to assess ovarian cancer risk among participants to distinguish high-risk and low-risk individuals and subsequently observe patterns in purchase history stratified by risk levels.

The questionnaire also includes questions about the symptoms experienced (if any) and number of visits to the general practitioner in the year leading up to cancer referral or diagnosis which were adopted from the proof-of-concept study. The final section of the questionnaire contains questions about which food and pharmacy stores participants shop at

For peer review only - http://bmjopen.bmj.com/site/about/guidelines.xhtml

regularly, at which stores they own a loyalty card, and frequency of use of those loyalty cards.

Clinical Questionnaire (Cases only)

Women with an ovarian cancer diagnosis will also have a clinical questionnaire completed by their healthcare team. This will provide the objective timeline and pathway for diagnosis (e.g. GP referral, emergency presentation, other), details on the type, stage, grade of ovarian cancer at diagnosis, surgical outcome if any, and BRCA status if the participant has been tested. We will use this information in risk assessment and to stratify the loyalty card data of cases and controls based on the time of diagnosis, and observe purchase history stratified by type, stage, and grade of ovarian cancer diagnoses.

Data collection processes (see figure 3)

In the next section we will describe, how we will be collecting and processing data in the CLOCS case-control study.

1) Data Acquisition

Upon choosing to participate, both cases and controls will need to complete a consent form to participate in CLOCS and return it to the CLOCS research team either by post (cases only) or the CLOCS website (controls only). The CLOCS consent form includes two sections. The first section details consent to take part in CLOCS, and the second section is for participants to consent to the CLOCS research team to request their data from high street retailers on their behalf. The consents for loyalty cards include the exact name of the individual and the exact membership number as written on their card. All individual consents including scanned copies of paper consents will be saved in the secure and encrypted server based in the "Secure Enclave" at Imperial College London. Participant loyalty card details will be entered in to a comma-separated values (CSV) file and encrypted for secure data request from retailers using a secure file transfer protocol (SFTP).

2) Data Extraction from the high street retailers

The CLOCS team will send the CSV file that contains a unique study ID, name of the individual and the exact loyalty card number specific to the retailer using the secure file transfer. The respective high street retailer will validate the participant information from the consent form with their loyalty card data. If loyalty card details cannot be validated,

For peer review only - http://bmjopen.bmj.com/site/about/guidelines.xhtml

the consents and any information from those individuals who have not consented to be re-contacted will be excluded from the study and deleted immediately. High street retailers will then generate a dataset for all consenting individuals and will share the past purchase data with the CLOCS team using the SFTP. Depending on the high street retailer's records and when the participant obtained the loyalty card, up to 7 years of participants' past purchasing data prior to the date of recruitment will be shared.

3) Data Merge

The CLOCS team will receive the encrypted participant purchase history from the high street retailers through the SFTP, save, and store all data to a secure and encrypted server based in the Secure Enclave at Imperial College London, described in *Data management*. All data will be pseudonymised and analysed on the secure server.

4) Data Deletion

The information relating to the participants' consents for providing past purchase data will be retained in line with each high street retailers' data retention policy. After 10 years, all paper-based documents will be shredded and destroyed in accordance with Imperial College London's data deletion and retention processes. During the lifetime of the study, participants are given the option to withdraw consent which will mean that all data associated with that individual will be deleted and records will be removed from CLOCS.

Sample size and statistical power

Based on the modelled data from the pilot study[13], we hypothesise that we could detect a difference in purchase proportions of 8.3% at 3 months prior to diagnosis, 6.3% at 6 months, 4.3% at 9 months and 2.3% at 12 months prior to diagnosis between cases and controls. Using the two-sample test for proportions the minimum group size to detect these differences at 80% power and $\alpha=0.05$ would be 125 (-3 months), 185 (-6 months), 328 (-9 months) and 969 (-12 months). We modelled the power versus time prior to diagnosis and chose 10 months as our target to retain sufficient power for larger effect sizes at earlier time points. Therefore, in the final analysis we will have >80% power at $\alpha=0.05$ to detect an increase in proportion of purchases in cases (>4.7%) vs controls (1.4%) at 10 months prior to diagnosis, based on the modelled data, with a minimum n=431 cases. We aim to recruit 500 participants to account for 10-15% of very low frequency card users. We will compare proportions using

For peer review only - http://bmjopen.bmj.com/site/about/guidelines.xhtml

Fishers Exact Test comparing target and non-target purchases in cases at each time point prior to diagnosis compared with the monthly proportions in the controls. We will report proportions, 95% confidence intervals and *P*-values.

Statistical Methods

We will use descriptive statistics to provide details on the characteristics of the cases and controls by sociodemographic (e.g. age, ethnicity) and by risk factors (e.g. parity, oral contraceptive use, etc.). We will report feasibility factors such as number of people who had both, or one loyalty card, and number of years of data included in the study.

Cases and controls will be matched 1:1 by age (5-year age groups) and ethnicity. We will aim to define the time by which cases and controls become statistically significantly different in their purchase behaviours using a multivariable, conditional logistic regression model with ovarian cancer as the outcome and "purchase variables" at different time points to diagnosis as the exposure, adjusting for stage and histology and other potentially confounding variables collected from the risk factor questionnaire. We will compare the proportions of purchases in cases to controls, stratified by risk quintiles defined by the risk model (estimated n=86 per quintile) or by histology (with expected proportions of high-grade serous, mucinous cases, endometrioid, clear cell, and low-grade serous cases). Established risk factors have been reported to modestly discriminate between cases and controls (AUC=0.66)[15], and polygenic risk scores only slightly improves these performances. This model has however been proposed to improve epithelial ovarian cancer detection with risk stratified screening[16]. We will estimate the predictive values of this "early diagnosis" test for each stratum.

We will conduct exploratory analyses investigating other methods leveraging the auto correlation in the longitudinal data. Specifically, we will explore the evolutions of individual frequency of purchases and purchase trajectories, to identify purchase patterns (e.g. increasing purchases before diagnosis, purchase peaks, etc.), that are indicative/predictive of the disease onset and of the time-to-onset.

All analyses will be done using the statistical software R.

Interim Analysis

An interim analysis will be conducted after six months of CLOCS recruitment to provide an initial exploratory analysis of the data and assess the demographics of CLOCS participants. This will help determine whether recruitment will need to target specific demographics (e.g.

For peer review only - http://bmjopen.bmj.com/site/about/guidelines.xhtml

age groups) in order to ensure there is a representative distribution of ages and ethnicities for the matched analysis.

Study risks and sources of bias

There are no clear risks for participants in the CLOCS. However, it may be emotional or distressing for patients to think about cancer and the first time they noticed some changes in their body (i.e. symptoms). The CLOCS team intended to design the survey questions in the most sensible and sensitive way to ensure that there are no negative effects of this study on patients' well-being. All participants are advised to see a medical professional in the information sheet if they are worried about a symptom or sign that they might have been experiencing.

For many loyalty card holders, purchases will be for themselves and other individuals in their household. The CLOCS team will not be able to quantify what purchases are consumed by the participant and by other members of the household, but the team will be able to make assumptions to adjust accordingly.

The CLOCS team acknowledges that not all participants will have used their loyalty card with every purchase for various reasons (e.g. card not with them at time of purchase, in a hurry, etc.) and that participants will have made purchases at other shops not recorded with their loyalty cards observed in this study. For this reason, the risk factor questionnaire asks participants to indicate how often they use their loyalty card when shopping, at which stores they shop most regularly and own a loyalty card, so that these can be taken account at the analysis stage.

In any retrospective case-control study, there is the potential for recall bias. Participants with ovarian cancer could report experiencing more symptoms more frequently than may have occurred, but for those who report they self-managed these symptoms, the CLOCS team will be able to observe loyalty card purchase data to validate the amount and frequency of self-medication.

Data management

The CLOCS research team will act as *data processors* when requesting data from high street retailers and the high street retailers would be *data controllers*. CLOCS will become the data controller for the self-reported questionnaire data and the clinical questionnaire data. The consenting participants will be *data owners* and will have the right to withdraw their consent from the study at any time without giving any reason to the study team.

For peer review only - http://bmjopen.bmj.com/site/about/guidelines.xhtml

Only the CLOCS research team will have access to the paper-based consent forms which will be kept in a locked cupboard and server in a locked room and secure building that requires security badge access to enter. The data will be analysed in a secure environment with access limited to the research team. Participants will be given a unique study ID barcode which means all datasets will be pseudonymised.

All data will be stored and processed in Imperial College's ISO27001 certified secure environment – the "Secure Enclave". This is a fully managed infrastructure and secure environment providing high availability, resilience and business continuity through multiple servers, back-ups and disaster recovery measures. A robust data security model has been designed to protect sensitive personal and medical data from the potential risk of unauthorised access or distribution. No one other than the research team will have access to the research dataset.

The CLOCS website is compliant with the high level of data security required by Imperial College London and General Data Protection Regulation (GDPR). The data collected through the website only resides in the Secure Enclave and not in the browser or website. It also uses Public Key Encryption, which encrypts data from the site that can only be unlocked once within the enclave. These requirements are equalled by support for the user to complete the online survey, by using content and interfaces that are easy to use, especially on mobile phones.

We have strict data processing agreements in place with our commercial partners to ensure that data is protected at all times. Commercial partners will not have access to the study data and will not financially benefit from this project.

Patient and public involvement

Ovarian cancer patients and members of the public have been involved in the development of the CLOCS from the beginning, participating in the proof-of-concept study that assessed the general public's acceptability of the use of commercial and health data linkages for cancer symptom surveillance[13]. Two ovarian cancer patient advocates who participated in the pilot study have taken part in research meetings developing this case-control study and reviewed study documentation. They have been and will continue to be involved in ongoing development of CLOCS methods and dissemination of the research results through social media, media outlets and patient groups. The questionnaire for women without ovarian cancer has also been reviewed and approved by members of the public.

For peer review only - http://bmjopen.bmj.com/site/about/guidelines.xhtml

We also use our website for providing information to the patients and public. For instance, all research goals, how we will be analysing data, confidentiality and data security are all detailed for the lay population. There is an animation hosted on our website that is aiming to explain the study in simpler words. All materials are reviewed and approved by patient advocates. We carry out annual research meetings with stakeholders including research nurses, patients, academic collaborators, retailers, funders and public to provide updates and receive feedback on this project.

ETHICS AND DISSEMINATION

CLOCS was reviewed and approved by the North West - Greater Manchester South Research Ethics Committee (19/NW/0427). The study is registered as an NHIR portfolio study (Ref: CPMS 43323), with the ISRCTN (14897082), and is registered on clinicaltrials.gov (NCT03994653).

We will disseminate the outcomes of the study using academic publications, the study website (clocsproject.org.uk), social media (@CLOCS_Imperial), and send a report to the research sites that supported the study once the results are published. We will aim to publish the results in a peer-reviewed journal in accordance with the STROBE statement for case-control studies[17]. We will also use the study website to inform the public about the study outcomes.

Data Sharing Policy

All access to the CLOCS data collected from the retailers will be restricted and will not be shared with other researchers due to the data sharing agreements with the retailers. However, we do encourage other researchers to contact us for collaborations. If they are interested, they are given the opportunity to review the metadata by directly contacting us and submit a research proposal where the data analyses will be carried out by a member of the CLOCS team. All proposals will be reviewed case by case by the PI and the rest of the research team.

For peer review only

For peer review only - http://bmjopen.bmj.com/site/about/guidelines.xhtml

Author Contributions:

JMF and YH conceived the study. HRB wrote the first draft of the manuscript. YH, JMF, SS and MC-H commented and reviewed the final draft of the manuscript. All authors agree on the final version of the protocol.

Funding statement:

Cancer Loyalty Card Study (CLOCS) is funded by Cancer Research UK (CRUK) Early Detection Project Grant (C38463/A26726). YH and HRB are funded by CRUK.

Competing interests statement

The authors declare no competing interests.

Word Count: 3942

Acknowledgements

We thank NDP Studio Limited for their support and development of the study website (www.clocsproject.org.uk). We thank our patient and public representatives for reviewing the study materials and providing guidance on study acceptability from the conception of CLOCS to data collection. The authors acknowledge infrastructure and recruitment support from the Imperial Experimental Cancer Medicine Centre, Cancer Research UK Imperial Centre, the National Institute for Health Research Imperial Biomedical Research Centre, and the Ovarian Cancer Action Research Centre. We thank Boots UK employees who facilitated the project for their support and guidance during the development of this project. We thank Eric Johnson for guidance on data security and GDPR.

Figure Legends

Figure 1: Pathway to diagnosis highlighting the patient interval that CLOCS will investigate.

Figure 2: CLOCS recruitment process.

Figure 3: CLOCS data flowchart.

For peer review only - http://bmjopen.bmj.com/site/about/guidelines.xhtml

REFERENCES

1 Cancer Research UK. Ovarian cancer statistics. https://www.cancerresearchuk.org/health-professional/cancer-statistics/statistics-by-cancer-type/ovarian-cancer (accessed 9 Apr 2019).

2 Funston G, Van Melle M, Baun M, *et al.* Variation in the initial assessment and investigation for ovarian cancer in symptomatic women: A systematic review of international guidelines. BMC Cancer. 2019. doi:10.1186/s12885-019-6211-2

3 National Institute for Health and Care Excellence. Ovarian cancer: recognition and initial management. Clin. Guidel. [CG122]. 2011.https://www.nice.org.uk/guidance/cg122/chapter/1-Guidance#detection-in-primary-care (accessed 26 Nov 2019).

4 Ebell M, Culp M, Radke T. A Systematic Review of Symptoms for the Diagnosis of Ovarian Cancer. Am. J. Prev. Med. 2016;**50**:384–94. doi:10.1016/j.amepre.2015.09.023

5 Goff B. Symptoms associated with ovarian cancer. *Clin Obstet Gynecol* 2012;**55**:36–42. doi:10.1097/GRF.0b013e3182480523

6 Brain K, Smits S, Simon A, *et al.* Ovarian cancer symptom awareness and anticipated delayed presentation in a population sample. *BMC Cancer* Published Online First: 2014. doi:10.1186/1471-2407-14-171

7 Low E, Waller J, Menon U, *et al.* Ovarian cancer symptom awareness and anticipated time to help-seeking for symptoms among UK women. *J Fam Plan Reprod Heal Care* Published Online First: 2013. doi:10.1136/jfprhc-2012-100473

8 ICBP. The international cancer benchmarking partnership evidence for policy and practice October 2016. 2016.

9 Low E, Whitaker K, Simon A, *et al.* Women's interpretation of and responses to potential gynaecological cancer symptoms: A qualitative interview study. *BMJ Open* 2015;**5**. doi:10.1136/bmjopen-2015-008082

10 Whitaker K, Winstanley K, Macleod U, *et al.* Low cancer suspicion following experience of a cancer 'warning sign'. *Eur J Cancer* 2015;**51**:2473–9. doi:10.1016/j.ejca.2015.07.014

11 Jayde V, Boughton M. The diagnostic journey of ovarian cancer: A review of the literature and suggestions for practice. Contemp. Nurse. 2012. doi:10.5172/conu.2012.41.1.5

12 Gilbert L, Basso O, Sampalis J, *et al.* Assessment of symptomatic women for early

For peer review only - http://bmjopen.bmj.com/site/about/guidelines.xhtml

diagnosis of ovarian cancer: results from the prospective DOvE pilot project. *Lancet Oncol* 2012;**13**:285–91. doi:10.1016/S1470-2045(11)70333-3

13 Flanagan J, Skrobanski H, Shi X, *et al.* Self-Care Behaviors of Ovarian Cancer Patients Before Their Diagnosis: Proof-of-Concept Study. *JMIR Cancer* 2019;**5**:e10447. doi:10.2196/CANCER.10447

14 Reid B, Permuth J, Sellers T. Epidemiology of ovarian cancer: a review. *Cancer Biol Med* 2017;**14**:9–32. doi:10.20892/j.issn.2095-3941.2016.0084

15 Clyde M, Palmieri Weber R, Iversen E, *et al.* Risk Prediction for Epithelial Ovarian Cancer in 11 United States-Based Case-Control Studies: Incorporation of Epidemiologic Risk Factors and 17 Confirmed Genetic Loci. *Am J Epidemiol* 2016;**184**:555–69. doi:10.1093/aje/kww091

16 Meisel SF, Side L, Fraser L, *et al.* Population-based, risk-stratified genetic testing for ovarian cancer risk: A focus group study. *Public Health Genomics* 2013;**16**:184–91. doi:10.1159/000352028

17 von Elm E, Altman DG, Egger M, *et al.* The Strengthening the Reporting of Observational Studies in Epidemiology (STROBE) statement: guidelines for reporting observational studies. *Lancet* Published Online First: 2007. doi:10.1016/S0140-6736(07)61602-X

For peer review only

For peer review only - http://bmjopen.bmj.com/site/about/guidelines.xhtml

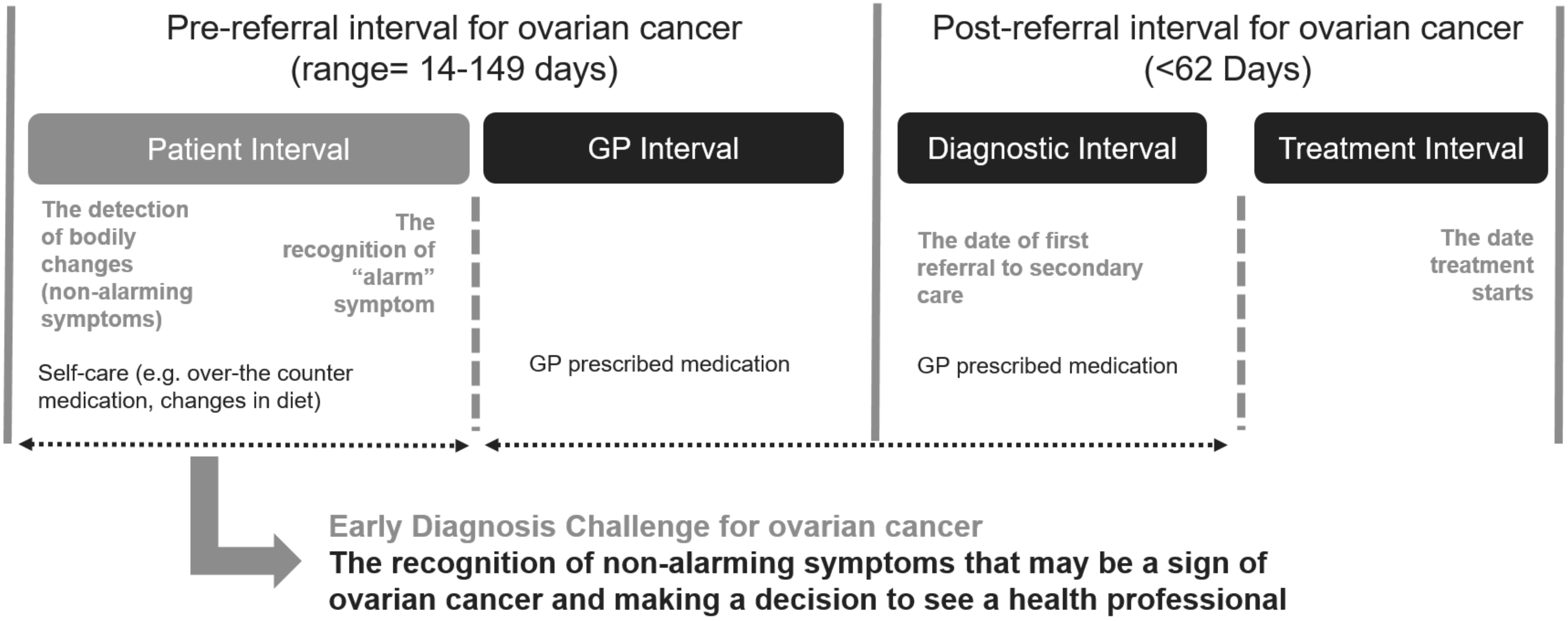
Pre-referral interval for ovarian cancer
(range= 14-149 days)
Post-referral interval for ovarian cancer
(<62 Days)
Patient Interval
GP Interval
Diagnostic Interval
Treatment Interval
The detection of bodily changes (non-alarming symptoms)
The recognition of "alarm" symptom
The date of first referral to secondary care
The date treatment starts
Self-care (e.g. over-the counter medication, changes in diet)
GP prescribed medication
GP prescribed medication
Early Diagnosis Challenge for ovarian cancer
The recognition of non-alarming symptoms that may be a sign of ovarian cancer and making a decision to see a health professional

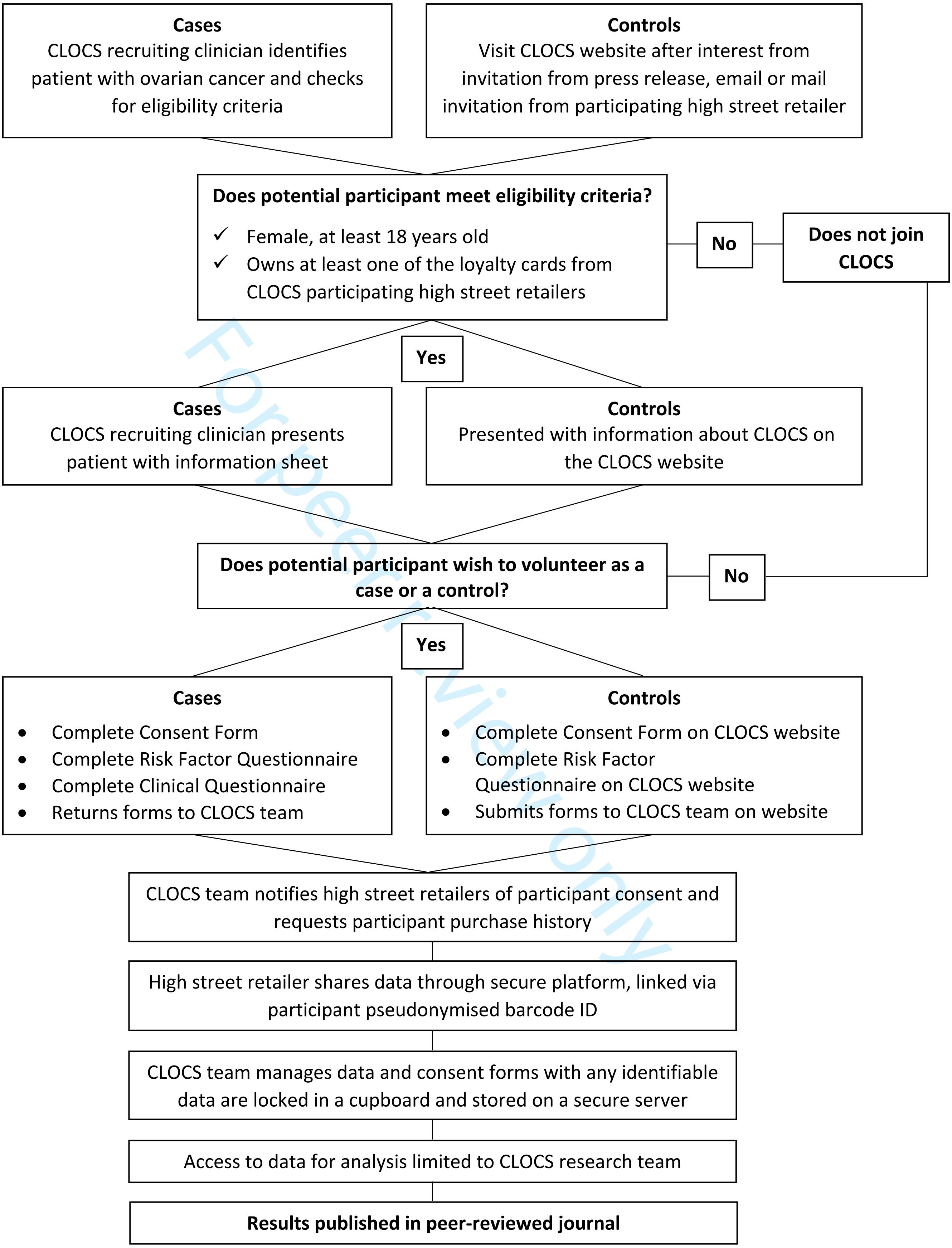

For peer review only

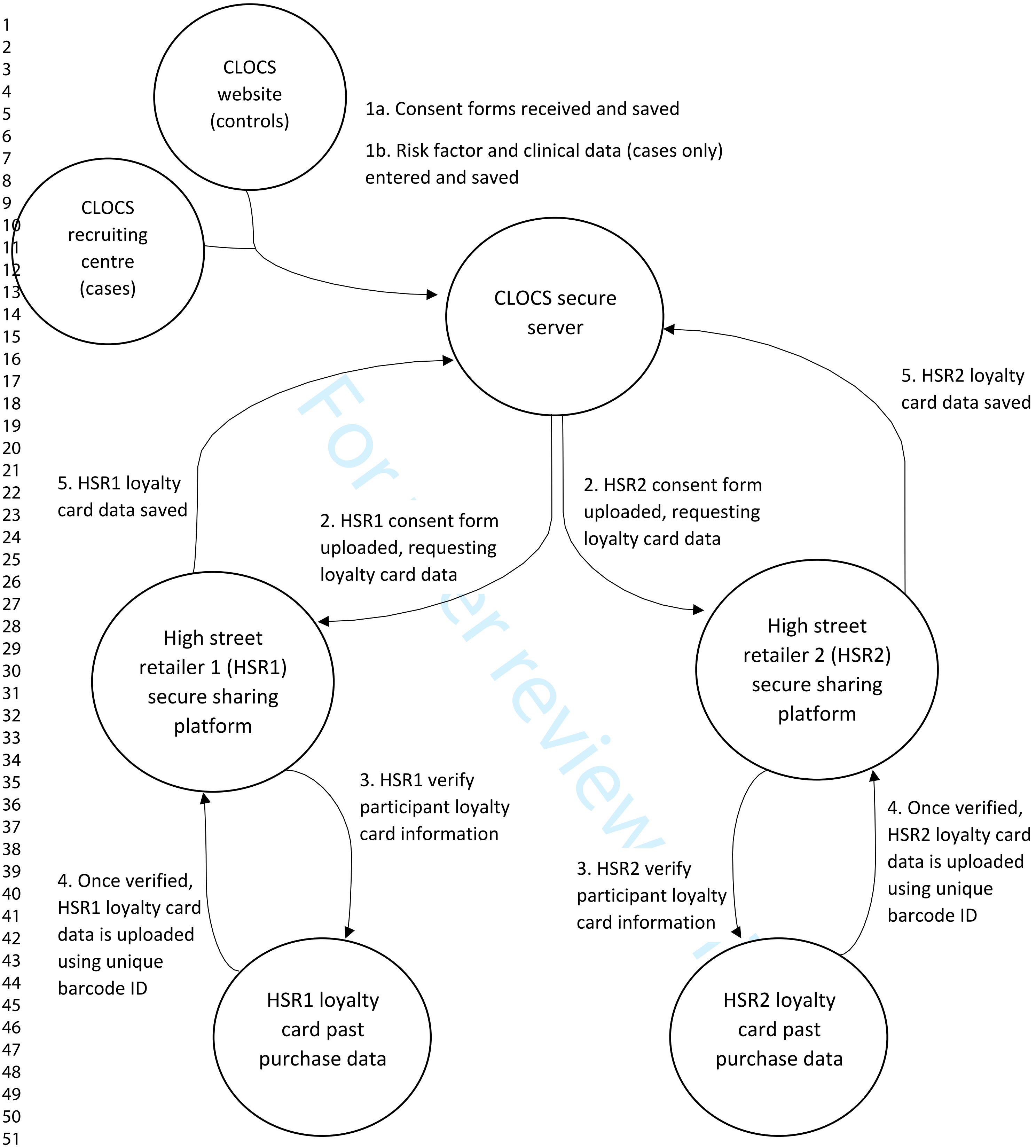
CLOCS website (controls)
CLOCS recruiting centre (cases)
1a. Consent forms received and saved
1b. Risk factor and clinical data (cases only) entered and saved
CLOCS secure server
5. HSR2 loyalty card data saved
5. HSR1 loyalty card data saved
2. HSR1 consent form uploaded, requesting loyalty card data
2. HSR2 consent form uploaded, requesting loyalty card data
High street retailer 1 (HSR1) secure sharing platform
High street retailer 2 (HSR2) secure sharing platform
3. HSR1 verify participant loyalty card information
3. HSR2 verify participant loyalty card information
4. Once verified, HSR1 loyalty card data is uploaded using unique barcode ID
4. Once verified, HSR2 loyalty card data is uploaded using unique barcode ID
HSR1 loyalty card past purchase data
HSR2 loyalty card past purchase data

BMJ Open

Cancer Loyalty Card Study (CLOCS): protocol for an observational case-control study focusing on the patient interval in ovarian cancer diagnosis

Journal:	*BMJ Open*
Manuscript ID	bmjopen-2020-037459.R1
Article Type:	Protocol
Date Submitted by the Author:	30-Apr-2020
Complete List of Authors:	Brewer, Hannah; Imperial College London, Surgery and Cancer<br>Hirst, Yasemin; University College London, Epidemiology and Public Health<br>Sundar, Sudha; University of Birmingham, Cancer Sciences<br>Chadeau-Hyam, Marc; Imperial College London, MRC Centre for Environment and Health; Imperial College London, Epidemiology and Biostatistics, School of Public Health<br>Flanagan, James; Imperial College London, Surgery and Cancer
**Primary Subject Heading**:	Oncology
Secondary Subject Heading:	Epidemiology, Public health
Keywords:	ONCOLOGY, Gynaecological oncology < GYNAECOLOGY, Epidemiology < ONCOLOGY, PUBLIC HEALTH

SCHOLARONE™
Manuscripts

I, the Submitting Author has the right to grant and does grant on behalf of all authors of the Work (as defined in the below author licence), an exclusive licence and/or a non-exclusive licence for contributions from authors who are: i) UK Crown employees; ii) where BMJ has agreed a CC-BY licence shall apply, and/or iii) in accordance with the terms applicable for US Federal Government officers or employees acting as part of their official duties; on a worldwide, perpetual, irrevocable, royalty-free basis to BMJ Publishing Group Ltd ("BMJ") its licensees and where the relevant Journal is co-owned by BMJ to the co-owners of the Journal, to publish the Work in this journal and any other BMJ products and to exploit all rights, as set out in our licence.

The Submitting Author accepts and understands that any supply made under these terms is made by BMJ to the Submitting Author unless you are acting as an employee on behalf of your employer or a postgraduate student of an affiliated institution which is paying any applicable article publishing charge ("APC") for Open Access articles. Where the Submitting Author wishes to make the Work available on an Open Access basis (and intends to pay the relevant APC), the terms of reuse of such Open Access shall be governed by a Creative Commons licence – details of these licences and which Creative Commons licence will apply to this Work are set out in our licence referred to above.

Other than as permitted in any relevant BMJ Author's Self Archiving Policies, I confirm this Work has not been accepted for publication elsewhere, is not being considered for publication elsewhere and does not duplicate material already published. I confirm all authors consent to publication of this Work and authorise the granting of this licence.

For peer review only

TITLE

Cancer Loyalty Card Study (CLOCS): protocol for an observational case-control study focusing on the patient interval in ovarian cancer diagnosis

Short Title

Cancer Loyalty Card Study (CLOCS): protocol for a case-control study

AUTHORS

Hannah R. Brewer[1], Yasemin Hirst[2], Sudha Sundar[3,4], Marc Chadeau-Hyam[5,6] and James M. Flanagan[1]

1. Department of Surgery and Cancer, Imperial College London, London, UK
2. Department of Behavioural Science and Health, University College London, London, UK
3. Pan-Birmingham Gynaecological Cancer Centre, City Hospital, Birmingham, UK.
4. Institute of Cancer and Genomic Sciences, University of Birmingham, Birmingham, UK.
5. MRC Centre for Environment and Health, Imperial College London, London, United Kingdom.
6. Department of Epidemiology and Biostatistics, School of Public Health, Imperial College London, London, United Kingdom.

Address for Correspondence:

Dr. James M. Flanagan

Division of Cancer, Department of Surgery and Cancer,

Faculty of Medicine, Imperial College London

4th Floor IRDB, Hammersmith Campus, Du Cane Road, London W12 0NN

Tel: 020 75941804; email: j.flanagan@imperial.ac.uk

For peer review only - http://bmjopen.bmj.com/site/about/guidelines.xhtml

ABSTRACT

Introduction

Ovarian Cancer is the eighth most common cancer in women worldwide, and about one in five women with ovarian cancer do not receive treatment, because they are too unwell by the time they are diagnosed. Symptoms of ovarian cancer are non-specific or can be associated with other common conditions, and women experiencing these symptoms have been shown to self-manage them using over-the-counter medication. Results from a recent proof of concept study suggest there may be an increase in the purchases of pain killers and indigestion medication 10-12 months before ovarian cancer diagnosis. We propose a case-control study, as part of a larger project called the Cancer Loyalty Card Study (CLOCS), to investigate whether a significant change in medication purchases could be an indication for early signs of ovarian cancer, using data already collected through store loyalty cards.

Methods and Analysis

Using a retrospective case-control design, we aim to recruit 500 women diagnosed with ovarian cancer (cases) and 500 women without ovarian cancer (controls) in the United Kingdom who hold a loyalty card with at least one participating high street retailer (i.e. all shops accessible within a postcode sector). We will use pre-existing loyalty card data to compare past purchase patterns of cases with those of controls. In order to assess ovarian cancer risk in participants and their purchase patterns, we will collect information from participants on ovarian cancer risk factors and clinical data including symptoms experienced before diagnosis from recruited women with ovarian cancer.

Ethics and dissemination

CLOCS was reviewed and approved by the North West - Greater Manchester South Research Ethics Committee (19/NW/0427). Study outcomes will be disseminated through academic publications, the study website, social media, and a report to the research sites that support the study once results are published.

Study Registration Numbers

CLOCS is registered with ISRCTN (14897082), NIHR portfolio (CPMS 43323), and clinicaltrials.gov (NCT03994653).

Keywords:

Ovarian cancer, early detection, purchase behaviour, observational case-control study, commercial data

For peer review only - http://bmjopen.bmj.com/site/about/guidelines.xhtml

Strengths and limitations of this study

- This study is a novel approach to investigate whether routinely collected commercial purchase data can be used to reduce delays in ovarian cancer diagnosis.
- Ovarian cancer advocates have been involved in the conception, design and conduct of the study.
- A key strength of the study is the detailed information available for purchases recorded for up to 7 years prior to enrolment.
- A key limitation is that loyalty card holders may not use their cards for every purchase, may shop at other stores not being accessed in this study and may be purchasing items for other family members.
- In general, due to the retrospective nature of case-control studies, there is potential for the misreporting of exposures in the risk factor questionnaire by cases and controls (recall bias).

For peer review only

INTRODUCTION

Approximately 7,200 new cases of ovarian cancer are diagnosed each year in the UK, and with over 4,200 women dying from the disease each year, it is a particularly lethal form of cancer[1]. Diagnosing ovarian cancer in primary and secondary care settings among symptomatic patients is a challenge due to their low positive predictive value of symptoms and symptom presentation at various stages of the ovarian cancer prognosis[2, 3]. Symptoms, such as increased abdominal size, pelvic pain, abdominal pain and bloating, feeling full quickly and difficulty eating have been identified as high risk symptoms but equally it has been reported that the absence of a symptom does not help ruling out ovarian cancer[4, 5]. For patients whose symptoms do not warrant an immediate referral earlier presentation in primary care is crucial to reduce delays in diagnosis and subsequent treatment.

However, symptom recognition and help-seeking in primary care are also not without challenges. While in some cases patients may present very quickly, for some patients it could take up to 12 months before they seek any medical help[6]. It has been shown that greater awareness of symptoms alone does not facilitate help seeking for ovarian cancer because symptoms are often perceived as vague or trivial[7, 8]. The major reasons for not presenting to the primary care are often highlighted as “not wanting to waste the GP’s time”[9],

For peer review only - http://bmjopen.bmj.com/site/about/guidelines.xhtml

normalisation of these symptoms[10], and few women believing that their symptom(s) might be a sign of cancer[11]. Women might choose to self-manage such symptoms by seeking advice from sources other than a primary care physician, e.g. pharmacists, family, internet, and using over-the-counter medication[12] which may also have been moderated by other factors such as access to healthcare, sociodemographic factors and other competing priorities, e.g. work and care responsibilities[7-11].

There are currently no organised screening programmes offered to women that could help detect ovarian cancer at an earlier stage, and there is little evidence for effective interventions aiming to improve symptom recognition and help-seeking.

Therefore, novel methods are needed to improve earlier detection of low-volume disease or earlier stage to improve survival[13]. A recent proof of concept study investigated whether purchase information collected through the high street retailers' loyalty cards (e.g. store points cards) could be used to identify women's self-care behaviours prior to presenting to health care professionals. This study included data from a major high street retailer's loyalty card of women who have previously been diagnosed with ovarian cancer and consented their past purchase data to be provided to the researchers. The study suggested that there may be an increase in the purchases of pain killers and indigestion tablets up to 10-12 months before diagnosis[14]. This proof-of-concept study provided a new model of investigation for symptom recognition and help-seeking among ovarian cancer patients. It highlighted potential strengths and limitations in its methodology for future research. Building on these results, self-care behaviours of ovarian cancer patients will be further investigated with a case-control study within a larger project called Cancer Loyalty Card Study (CLOCS). CLOCS hypothesises that a significant change in purchasing behaviour, primarily an increase in self-medication or self-care behaviours to treat symptoms prior to presentation in primary care (see Figure 1), could be an indication for early signs of ovarian cancer.

Study aims

The primary aim of this case-control study is to investigate pre-diagnosis purchasing behaviours of ovarian cancer patients (cases) with women without a previous ovarian cancer diagnosis (controls) using data collected through two major high street retailers' loyalty card schemes. We will investigate purchase behaviour of items that are suspected to treat symptoms of ovarian cancer (e.g. painkillers, indigestion tablets) as well as a change in frequency or quantity of other purchased items. The secondary aim of CLOCS will be to

For peer review only - http://bmjopen.bmj.com/site/about/guidelines.xhtml

investigate whether or not a purchase threshold as an “alert” about potential ovarian cancer symptoms in individuals can be possible using loyalty card data and determining the predictive utility of purchasing behaviours in the early detection of ovarian cancer based on ovarian cancer risk. This would be important to inform if future interventions would be possible using loyalty card data.

METHODS AND ANALYSIS

Study design and setting

CLOCS will use a retrospective case-control design to compare past purchase patterns of women with and without ovarian cancer using already existing data gathered by two major high street retailers (i.e. retailers that can be accessed by the general population in all precincts). Furthermore, we will collect data on risk factors for ovarian cancer, symptoms experienced before diagnosis and clinical data from ovarian cancer patients using self-reported questionnaires.

CLOCS will be recruiting across the United Kingdom from at least 40 National Health Service (NHS) Trusts and Hospitals and recruit healthy volunteers using online recruitment.

Study timeline

The CLOCS case-control study will take place from 1st November 2019 until 1st February 2022.

Participants

Inclusion/Exclusion Criteria

Cases

Women, at least 18 years old, diagnosed and living with ovarian cancer (including high-grade serous, low-grade serous, endometrioid, clear cell, mucinous, borderline, and other subtypes) within the previous 2 years from the recruitment date who own at least one participating high street retailer loyalty card are eligible to join CLOCS case-control study as cases.

There are no further clinical criteria for patients to be excluded from this study. Patients’ usual care will continue to be provided by the health care professionals, and CLOCS participation does not exclude patients from participating in other clinical or non-clinical studies.

Controls

For peer review only - http://bmjopen.bmj.com/site/about/guidelines.xhtml

Women, at least 18 years old, who have not been diagnosed with ovarian cancer and own at least one of the loyalty cards from the participating retailers are eligible to join the CLOCS as controls. We are not excluding women with previous cancer diagnosis, or women who may have had their ovaries removed for reasons other than cancer; however, these will be considered when cases are matched with controls.

Recruitment

The CLOCS case-control study recruitment process is outlined in figure 2 and described further detail below.

Cases

Eligible ovarian cancer patients will be recruited by members of their healthcare teams at approximately 40 NHS Trusts and Hospitals across the United Kingdom that have agreed to approach patients at clinics. These healthcare teams are registered as research sites and will have a principal investigator (PI) leading the recruitment to CLOCS. The PI and the research nurses will facilitate recruitment by identifying patients when they think it is suitable to approach patients based on patients' overall well-being at a suitable time (e.g. at waiting areas, during chemotherapy sessions), then will provide patients with the study information to consider participation. Patients will be given a paper-based participant pack that includes study consent form including consents for providing access to data collected by the retailers, self-report questionnaire, and a clinical questionnaire to be completed by the clinical staff facilitating the recruitment. If they wish to participate, patients can complete all the study documents at the recruitment site and post it back to the research team using the free-post envelope provided in the study pack, or they are also given the option to consider participation at home provided that the clinical questionnaire has been completed by a member their clinical team. The research sites will not need to record any data from the participants. The data collection from the cases will be a one-off process and no further contact with the patient is required for the study.

Controls

The control participants to the study will be recruited through the study website (www.clocsproject.org.uk) where data collection will be safeguarded using an encrypted web form that includes study consent and the self-report questionnaire. The website has been developed and maintained by NDP limited. The questionnaire for women without ovarian cancer will be confined to risk factors. The online participation will take up to 15 minutes and data will be transferred immediately to the secure CLOCS server (see figure 3). The

For peer review only - http://bmjopen.bmj.com/site/about/guidelines.xhtml

website approach was adopted as one of the outcomes of the proof-of-concept study. It aims to provide a transparent environment where potential participants can make informed decisions about participation in CLOCS, and the data risks are minimised using encryption. We have carefully designed the website to inform the general public about how we will process their data in CLOCS, how they can withdraw from the study if they wish to do so and use it as a platform to provide feedback as we progress in the larger CLOCS project. The research team will use several methods to recruit control participants to the study which will include a press release carried out by the project funders and the Imperial College London public engagement team, dissemination of study flyers and leaflets to patients in participating NHS recruitment clinics, and social media marketing techniques on Twitter and Facebook using paid advertisement and unpaid methods. The application of the social media recruitment methods will allow researchers to reach a larger cohort in the UK. The application of both paper-based and online recruitment methods will be carried out throughout CLOCS and will be recorded. The efficacy of these methods will be reported in peer-reviewed journals to inform online research recruitment to future case-control studies.

Data sources and variables included

Loyalty Card Data (For cases and controls)

CLOCS participants will hold a loyalty card at one or both participating retailers, including two health, beauty and/or grocery retailers that can be accessed by the UK population within all precincts allowing the study to assess purchases of painkillers and indigestion medication as well as eating habits. The study will not include prescription-based pharmacy data. Once participants consent for the CLOCS team to request their loyalty card data from the retailers on their behalf, the study will include individuals' past purchase data from up to six years prior to the date of study consent. The purchase data will include each individual purchase, date of purchase, the location (i.e. store postcode), and the product categorisation that is provided by the retailers. There will be no additional individual information recorded by the study team from this dataset and no additional individual information will be requested from the retailers. Please note that at this stage of the study, we will not be naming the high street retailers in publications and in our direct communications, but they are named to the potential participants when they agree to take part.

Ovarian Cancer Risk Questionnaire (For Cases and Controls)

In order to stratify the purchase differences between cases and controls based on ovarian cancer risk, we designed a questionnaire on ovarian cancer risk factors that will allow us to

For peer review only - http://bmjopen.bmj.com/site/about/guidelines.xhtml

determine individuals' ovarian cancer risk. Both cases and control participants will be asked to complete a short questionnaire about ethnicity, marital status, and the following well-established ovarian cancer risk factors: body mass index, age at menarche, menopausal status, age at menopause, parity, breastfeeding, hysterectomy, tubal ligation, cancer history, endometriosis, aspirin use, oral contraceptive use, hormone replacement therapy (HRT), family history of ovarian and breast cancers, vaping, and cigarette smoking[15]. These factors will be used to assess ovarian cancer risk among participants to distinguish high-risk and low-risk individuals and subsequently observe patterns in purchase history stratified by risk levels.

The questionnaire also includes questions about the symptoms experienced (if any) and number of visits to the general practitioner in the year leading up to cancer referral or diagnosis which were adopted from the proof-of-concept study[14].

The final section of the questionnaire contains questions about which food and pharmacy stores participants shop at regularly, at which stores they own a loyalty card, and frequency of use of those loyalty cards.

Clinical Questionnaire (Cases only)

Women with an ovarian cancer diagnosis will also have a clinical questionnaire completed by their healthcare team. This will provide the objective timeline and pathway for diagnosis (e.g. GP referral, emergency presentation, other), details on the type (high-grade serous, low-grade serous, endometrioid, clear cell, mucinous, borderline, and other), stage (I, II, III, or IV), grade of ovarian cancer at diagnosis (grade 1 or well differentiated, 2 or moderately differentiated, 3 or poorly differentiated/undifferentiated), surgical outcome if any (optimal debulking, sub-optimal debulking, or no surgery), and BRCA status if the participant has been tested (BRCA1 positive, BRCA2 positive, both negative, awaiting result). We will use this information in risk assessment and to stratify the loyalty card data of cases and controls based on the time of diagnosis, and observe purchase history stratified by type, stage, and grade of ovarian cancer diagnoses.

Data collection processes (see figure 3)

In the next section we will describe, how we will be collecting and processing data in the CLOCS case-control study.

1) *Data Acquisition*

For peer review only - http://bmjopen.bmj.com/site/about/guidelines.xhtml

Upon choosing to participate, both cases and controls will need to complete a consent form to participate in CLOCS and return it to the CLOCS research team either by post (cases only) or the CLOCS website (controls only). The CLOCS consent form includes two sections. The first section details consent to take part in CLOCS, and the second section is for participants to consent to the CLOCS research team to request their data from high street retailers on their behalf. The consents for loyalty cards include the exact name of the individual and the exact membership number as written on their card. All individual consents including scanned copies of paper consents will be saved in the secure and encrypted server based in the "Secure Enclave" at Imperial College London. Participant loyalty card details will be entered in to a comma-separated values (CSV) file and encrypted for secure data request from retailers using a secure file transfer protocol (SFTP).

2) Data Extraction from the high street retailers

The CLOCS team will send the CSV file that contains a unique study ID, name of the individual and the exact loyalty card number specific to the retailer using the secure file transfer. The respective high street retailer will validate the participant information from the consent form with their loyalty card data. If loyalty card details cannot be validated, the consents and any information from those individuals who have not consented to be re-contacted will be excluded from the study and deleted immediately. High street retailers will then generate a dataset for all consenting individuals and will share the past purchase data with the CLOCS team using the SFTP. Depending on the high street retailer's records and when the participant obtained the loyalty card, up to 7 years of participants' past purchasing data prior to the date of recruitment will be shared.

3) Data Merge

The CLOCS team will receive the encrypted participant purchase history from the high street retailers through the SFTP, save, and store all data to a secure and encrypted server based in the Secure Enclave at Imperial College London, described in *Data management*. All data will be pseudonymised and analysed on the secure server.

4) Data Deletion

The information relating to the participants' consents for providing past purchase data will be retained in line with each high street retailers' data retention policy. Ten years

For peer review only - http://bmjopen.bmj.com/site/about/guidelines.xhtml

after the study is complete, all paper-based documents will be shredded and destroyed in accordance with Imperial College London's data deletion and retention processes. Electronically held identifiable data including individual consent and contact details will be kept for 10 years after the study is complete and will be deleted from the secure server in accordance with Imperial College London's data deletion and retention processes. Past purchase data will be kept until the results are published or five years from the date of participation in the study, whichever comes sooner, before it will be anonymised and the link to all identifiable data will be deleted. During the lifetime of the study, participants are given the option to withdraw consent which will mean that all data associated with that individual will be deleted and records will be removed from CLOCS.

Sample size and statistical power

Based on the modelled data from the pilot study[14], we hypothesise that we could detect a difference in purchase proportions of 8.3% at 3 months prior to diagnosis, 6.3% at 6 months, 4.3% at 9 months and 2.3% at 12 months prior to diagnosis between cases and controls. Using the two-sample test for proportions the minimum group size to detect these differences at 80% power and $\alpha=0.05$ would be 125 (-3 months), 185 (-6 months), 328 (-9 months) and 969 (-12 months). We modelled the power versus time prior to diagnosis and chose 10 months as our target to retain sufficient power for larger effect sizes at earlier time points. Therefore, in the final analysis we will have >80% power at $\alpha=0.05$ to detect an increase in proportion of purchases in cases (>4.7%) vs controls (1.4%) at 10 months prior to diagnosis, based on the modelled data, with a minimum n=431 cases. We aim to recruit 500 participants to account for 10-15% of very low frequency card users. We will compare proportions using Fishers Exact Test comparing target (items suspected to be used to treat ovarian cancer symptoms) and non-target (all other items) purchases in cases at each time point prior to diagnosis compared with the average monthly proportions in the controls. We will report proportions, 95% confidence intervals and *P*-values.

Statistical Methods

We will use descriptive statistics to provide details on the characteristics of the cases and controls by sociodemographic (e.g. age, ethnicity) and by risk factors (e.g. parity, oral contraceptive use, etc.). We will report feasibility factors such as number of people who had both, or one loyalty card, and number of years of data included in the study.

For peer review only - http://bmjopen.bmj.com/site/about/guidelines.xhtml

Cases and controls will be matched 1:1 by age (5-year age groups) and ethnicity. Ethnicity will be matched based on the guidance from the Office for National Statistics[16]. We will aim to define the time by which cases and controls become statistically significantly different in their purchase behaviours using a multivariable, conditional logistic regression model with ovarian cancer as the outcome and "purchase proportion" at different time points to diagnosis as the exposure, adjusting for stage and histology and other potentially confounding variables collected from the risk factor questionnaire (including body mass index, age at menarche, menopausal status, age at menopause, parity, breastfeeding, hysterectomy, tubal ligation, cancer history, endometriosis, aspirin use, oral contraceptive use, hormone replacement therapy (HRT), family history of ovarian and breast cancers, vaping, and cigarette smoking[15]). We will compare the proportions of purchases in cases to controls, stratified by risk quintiles defined by the risk model (estimated n=86 per quintile) and by histology (with expected proportions of high-grade serous, mucinous cases, endometrioid, clear cell, and low-grade serous cases). Analyses will be performed for different types of medications separately and combined (e.g. a comparison of case-control purchase proportion of painkillers alone and indigestion medications alone, and then of painkillers and indigestion medications combined).

Established risk factors have been reported to modestly discriminate between cases and controls (AUC=0.66)[17], and polygenic risk scores only slightly improves these performances. This model has however been proposed to improve epithelial ovarian cancer detection with risk stratified screening[18]. We will estimate the predictive values of this "early diagnosis" test for each stratum.

We will conduct exploratory analyses investigating other methods leveraging the auto correlation in the longitudinal data. Specifically, we will explore the evolutions of individual frequency of purchases and purchase trajectories, to identify purchase patterns (e.g. increasing purchases before diagnosis, purchase peaks, etc.), that are indicative/predictive of the disease onset and of the time-to-onset.

All analyses will be done using the statistical software R.

Interim Analysis

An interim analysis will be conducted after six months of CLOCS recruitment to provide an initial exploratory analysis of the data and assess the demographics of CLOCS participants. This will help determine whether recruitment will need to target specific demographics (e.g.

For peer review only - http://bmjopen.bmj.com/site/about/guidelines.xhtml

age groups) in order to ensure there is a representative distribution of ages and ethnicities for the matched analysis.

Study risks and sources of bias

There are no clear risks for participants in the CLOCS. However, it may be emotional or distressing for patients to think about cancer and the first time they noticed some changes in their body (i.e. symptoms). The CLOCS team intended to design the survey questions in the most sensible and sensitive way to ensure that there are no negative effects of this study on patients' well-being. All participants are advised to see a medical professional in the information sheet if they are worried about a symptom or sign that they might have been experiencing.

For many loyalty card holders, purchases will be for themselves and other individuals in their household. The CLOCS team will not be able to quantify what purchases are consumed by the participant and by other members of the household, but the team will be able to make assumptions to adjust accordingly.

The CLOCS team acknowledges that not all participants will have used their loyalty card with every purchase for various reasons (e.g. card not with them at time of purchase, in a hurry, etc.) and that participants will have made purchases at other shops not recorded with their loyalty cards observed in this study. For this reason, the risk factor questionnaire asks participants to indicate how often they use their loyalty card when shopping, at which stores they shop most regularly and own a loyalty card, so that these can be taken account at the analysis stage.

It is possible that CLOCS participants could have other comorbidities, and their over-the-counter purchases might be used to treat symptoms from those instead of symptoms of ovarian cancer. However, many women with comorbidities are likely already receiving prescribed medication to treat these, and loyalty card data does not include information about prescriptions. Common comorbidities are expected to be present in similar frequencies among CLOCS participants with ovarian cancer and without ovarian cancer. The risk factor questionnaire will address some of these comorbidities, which will allow us to adjust for these in the analysis.

It is also possible that ovarian cancer patients may have already been seen by a health care professional for their symptoms and may have been prescribed medication for reasons other than ovarian cancer, e.g. urinary tract infection, irritable bowel syndrome, prior to being referred for suspected ovarian cancer. However, access to prescribed medication information would require access to primary care records and not included in this study. It would also

For peer review only - http://bmjopen.bmj.com/site/about/guidelines.xhtml

mean that patients have already sought help for their symptoms and the patient interval phase for symptom appraisal before help-seeking would have been finalised. We will control for this by adjusting for the self-reported number of visits to the GP leading up to the diagnosis. This would also be discussed as a potential limitation, and we will be cautious in our evaluations of the results if we observe a reduction in risk related purchases close to the date of diagnosis.

CLOCS limits participation to women who own a loyalty card from at least one of the participating retailers. Approximately 73% of women aged 50-80 years in the UK have a loyalty card to one of the participating high street retailers, and therefore the cardholders in CLOCS should be representative. Nevertheless, there will be selection bias against those who do not hold a loyalty card.

In any retrospective case-control study, there is the potential for recall bias. Participants with ovarian cancer could report experiencing more symptoms more frequently than may have occurred, but for those who report they self-managed these symptoms, the CLOCS team will be able to observe loyalty card purchase data to validate the amount and frequency of self-medication prior to diagnosis.

Data management

The CLOCS research team will act as *data processors* when requesting data from high street retailers and the high street retailers would be *data controllers*. CLOCS will become the data controller for the self-reported questionnaire data and the clinical questionnaire data. The consenting participants will be *data owners* and will have the right to withdraw their consent from the study at any time without giving any reason to the study team.

Only the CLOCS research team will have access to the paper-based consent forms which will be kept in a locked cupboard and server in a locked room and secure building that requires security badge access to enter. The data will be analysed in a secure environment with access limited to the research team. Participants will be given a unique study ID barcode which means all datasets will be pseudonymised.

All data will be stored and processed in Imperial College's ISO27001 certified secure environment – the "Secure Enclave". This is a fully managed infrastructure and secure environment providing high availability, resilience and business continuity through multiple servers, back-ups and disaster recovery measures. A robust data security model has been designed to protect sensitive personal and medical data from the potential risk of unauthorised access or distribution. No one other than the research team will have access to the research dataset.

For peer review only - http://bmjopen.bmj.com/site/about/guidelines.xhtml

The CLOCS website is compliant with the high level of data security required by Imperial College London and General Data Protection Regulation (GDPR). The data collected through the website only resides in the Secure Enclave and not in the browser or website. It also uses Public Key Encryption, which encrypts data from the site that can only be unlocked once within the enclave. These requirements are equalled by support for the user to complete the online survey, by using content and interfaces that are easy to use, especially on mobile phones.

We have strict data processing agreements in place with our commercial partners to ensure that data is protected at all times. Commercial partners will not have access to the study data and will not financially benefit from this project.

Patient and public involvement

Ovarian cancer patients and members of the public have been involved in the development of the CLOCS from the beginning, participating in the proof-of-concept study that assessed the general public's acceptability of the use of commercial and health data linkages for cancer symptom surveillance[14]. Two ovarian cancer patient advocates who participated in the pilot study have taken part in research meetings developing this case-control study and reviewed study documentation. They have been and will continue to be involved in ongoing development of CLOCS methods and dissemination of the research results through social media, media outlets and patient groups. The questionnaire for women without ovarian cancer has also been reviewed and approved by members of the public.

We also use our website for providing information to the patients and public. For instance, all research goals, how we will be analysing data, confidentiality and data security are all detailed for the lay population. There is an animation hosted on our website that is aiming to explain the study in simpler words. All materials are reviewed and approved by patient advocates. We carry out annual research meetings with stakeholders including research nurses, patients, academic collaborators, retailers, funders and public to provide updates and receive feedback on this project.

ETHICS AND DISSEMINATION

CLOCS was reviewed and approved by the North West - Greater Manchester South Research Ethics Committee (19/NW/0427). The study is registered as an NHIR portfolio study (Ref: CPMS 43323), with the ISRCTN (14897082), and is registered on clinicaltrials.gov

For peer review only - http://bmjopen.bmj.com/site/about/guidelines.xhtml

(NCT03994653). This paper describes the ethics approved protocol dated 8 August 2019 (version 2).

We will disseminate the outcomes of the study using academic publications, the study website (clocsproject.org.uk), social media (@CLOCS_Imperial), and send a report to the research sites that supported the study once the results are published. We will aim to publish the results in a peer-reviewed journal in accordance with the STROBE statement for case-control studies[19]. The STROBE statement we intend to use when reporting the results from this case-control study is listed in the supplementary material. We will also use the study website to inform the public about the study outcomes.

Data Sharing Policy

All access to the CLOCS data collected from the retailers will be restricted and will not be shared with other researchers due to the data sharing agreements with the retailers. However, we do encourage other researchers to contact us for collaborations. If they are interested, they are given the opportunity to review the metadata by directly contacting us and submit a research proposal where the data analyses will be carried out by a member of the CLOCS team. All proposals will be reviewed case by case by the PI and the rest of the research team.

For peer review only

Authors' contributions:

JMF and YH conceived the study. HRB wrote the first draft of the manuscript. YH, JMF, SS and MC-H commented and reviewed the final draft of the manuscript. All authors agree on the final version of the protocol.

Funding statement:

Cancer Loyalty Card Study (CLOCS) is funded by Cancer Research UK (CRUK) Early Detection Project Grant (C38463/A26726). YH and HRB are funded by CRUK.

Competing interests statement

The authors declare no competing interests.

Acknowledgements

We thank NDP Studio Limited for their support and development of the study website (www.clocsproject.org.uk). We thank our patient and public representatives for reviewing the study materials and providing guidance on study acceptability from the conception of CLOCS to data collection. The authors acknowledge infrastructure and recruitment support from the Imperial Experimental Cancer Medicine Centre, Cancer Research UK Imperial Centre, the National Institute for Health Research Imperial Biomedical Research Centre, and the Ovarian Cancer Action Research Centre. We thank Boots UK employees who facilitated the project for their support and guidance during the development of this project. We thank Eric Johnson for guidance on data security and GDPR.

Figure Legends

Figure 1: Pathway to diagnosis highlighting the patient interval that CLOCS will investigate.

Figure 2: CLOCS recruitment process.

Figure 3: CLOCS data flowchart.

For peer review only

REFERENCES

1. Cancer Research UK. Ovarian cancer statistics. https://www.cancerresearchuk.org/health-professional/cancer-statistics/statistics-by-cancer-type/ovarian-cancer. Accessed 9 Apr 2019.

2. Funston G, Van Melle M, Baun M, Jensen H, Helsper C, Emery J, et al. Variation in the initial assessment and investigation for ovarian cancer in symptomatic women: A systematic review of international guidelines. BMC Cancer. 2019.

3. National Institute for Health and Care Excellence. Ovarian cancer: recognition and initial management. Clinical guideline [CG122]. 2011.

4. Ebell M, Culp M, Radke T. A Systematic Review of Symptoms for the Diagnosis of Ovarian Cancer. American Journal of Preventive Medicine. 2016;50:384–94. doi:10.1016/j.amepre.2015.09.023.

5. Goff B. Symptoms associated with ovarian cancer. Clin Obstet Gynecol. 2012;55:36–42.

6. Hamilton W, Peters TJ, Bankhead C, Sharp D. Risk of ovarian cancer in women with symptoms in primary care: Population based case-control study. BMJ. 2009;339:616.

7. Brain K, Smits S, Simon A, Forbes L, Roberts C, Robbé I, et al. Ovarian cancer symptom awareness and anticipated delayed presentation in a population sample. BMC Cancer. 2014.

8. Low E, Waller J, Menon U, Jones A, Reid F, Simon A. Ovarian cancer symptom awareness and anticipated time to help-seeking for symptoms among UK women. J Fam Plan Reprod Heal Care. 2013.

9. ICBP. The international cancer benchmarking partnership evidence for policy and practice October 2016. 2016.

10. Low E, Whitaker K, Simon A, Sekhon M, Waller J. Women's interpretation of and responses to potential gynaecological cancer symptoms: A qualitative interview study. BMJ Open. 2015;5.

11. Whitaker K, Winstanley K, Macleod U, Scott S, Wardle J. Low cancer suspicion following experience of a cancer "warning sign." Eur J Cancer. 2015;51:2473–9.

12. Jayde V, Boughton M. The diagnostic journey of ovarian cancer: A review of the literature and suggestions for practice. Contemporary Nurse. 2012.

13. Gilbert L, Basso O, Sampalis J, Karp I, Martins C, Feng J, et al. Assessment of

For peer review only - http://bmjopen.bmj.com/site/about/guidelines.xhtml

For peer review only

symptomatic women for early diagnosis of ovarian cancer: results from the prospective DOvE pilot project. Lancet Oncol. 2012;13:285–91. doi:10.1016/S1470-2045(11)70333-3.

14. Flanagan J, Skrobanski H, Shi X, Hirst Y. Self-Care Behaviors of Ovarian Cancer Patients Before Their Diagnosis: Proof-of-Concept Study. JMIR Cancer. 2019;5:e10447. doi:10.2196/CANCER.10447.

15. Reid B, Permuth J, Sellers T. Epidemiology of ovarian cancer: a review. Cancer Biol Med. 2017;14:9–32. doi:10.20892/j.issn.2095-3941.2016.0084.

16. Office for National Statistics. Ethnic group, national identity and religion, Measuring equality: A guide for the collection and classification of ethnic group, national identity and religion data in the UK. 2016. https://www.ons.gov.uk/methodology/classificationsandstandards/measuringequality/ethnicgroupnationalidentityandreligion#toc.

17. Clyde M, Palmieri Weber R, Iversen E, Poole M, Doherty J, Goodman M, et al. Risk Prediction for Epithelial Ovarian Cancer in 11 United States-Based Case-Control Studies: Incorporation of Epidemiologic Risk Factors and 17 Confirmed Genetic Loci. Am J Epidemiol. 2016;184:555–69.

18. Meisel SF, Side L, Fraser L, Gessler S, Wardle J, Lanceley A. Population-based, risk-stratified genetic testing for ovarian cancer risk: A focus group study. Public Health Genomics. 2013;16:184–91.

19. von Elm E, Altman DG, Egger M, Pocock SJ, Gøtzsche PC, Vandenbroucke JP. The Strengthening the Reporting of Observational Studies in Epidemiology (STROBE) statement: guidelines for reporting observational studies. Lancet. 2007.

For peer review only

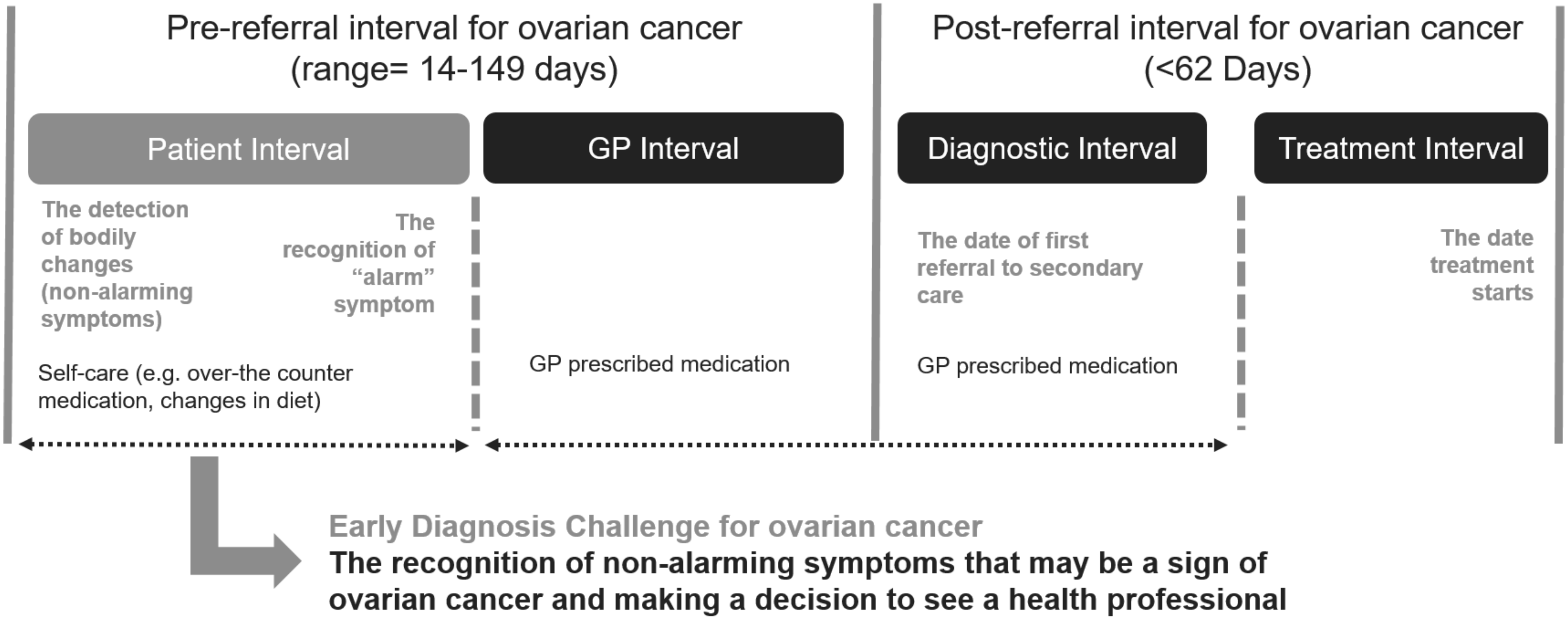
Pre-referral interval for ovarian cancer (range= 14-149 days)
Post-referral interval for ovarian cancer (<62 Days)
Patient Interval
GP Interval
Diagnostic Interval
Treatment Interval
The detection of bodily changes (non-alarming symptoms)
The recognition of "alarm" symptom
The date of first referral to secondary care
The date treatment starts
Self-care (e.g. over-the counter medication, changes in diet)
GP prescribed medication
GP prescribed medication
Early Diagnosis Challenge for ovarian cancer
The recognition of non-alarming symptoms that may be a sign of ovarian cancer and making a decision to see a health professional

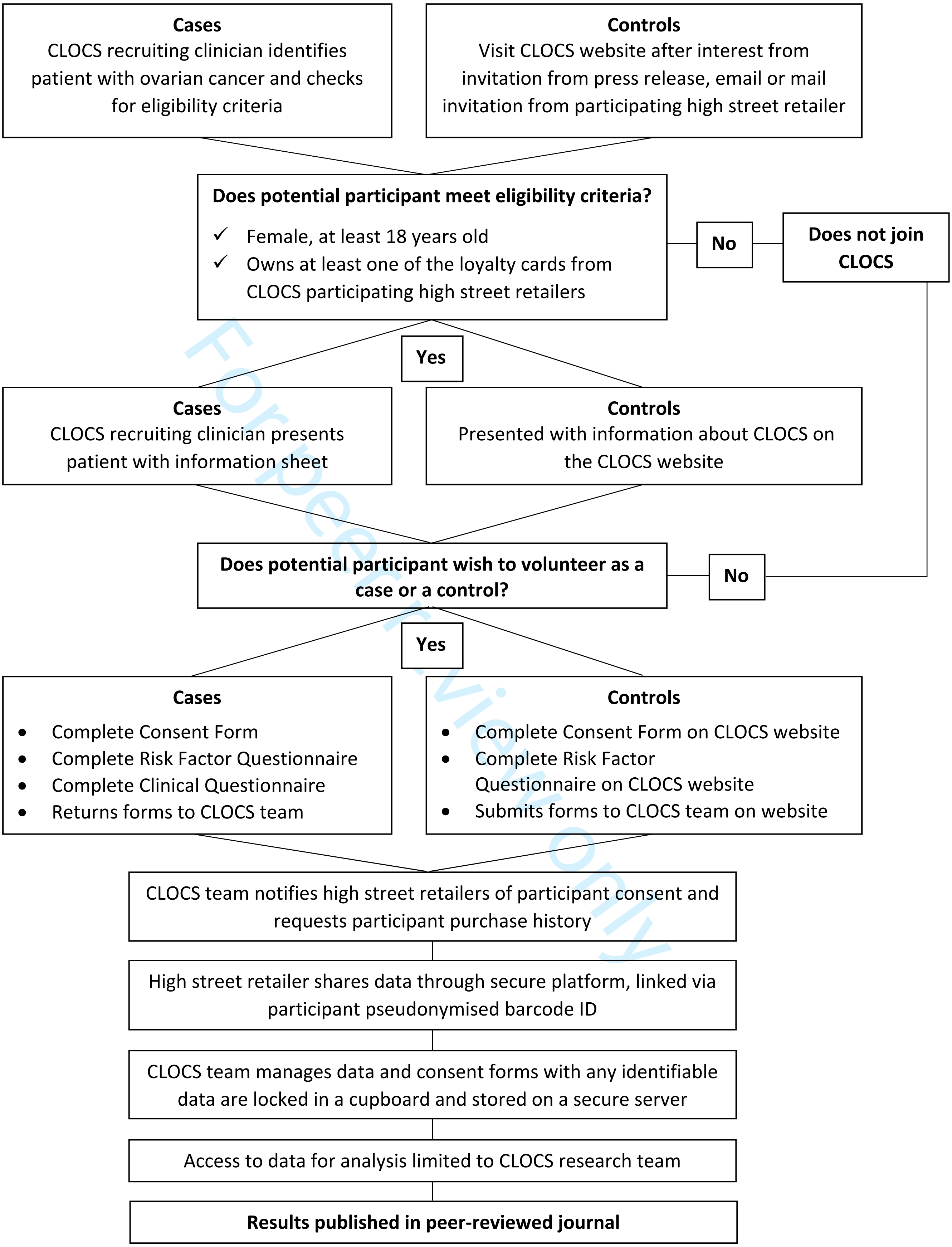

For peer review only

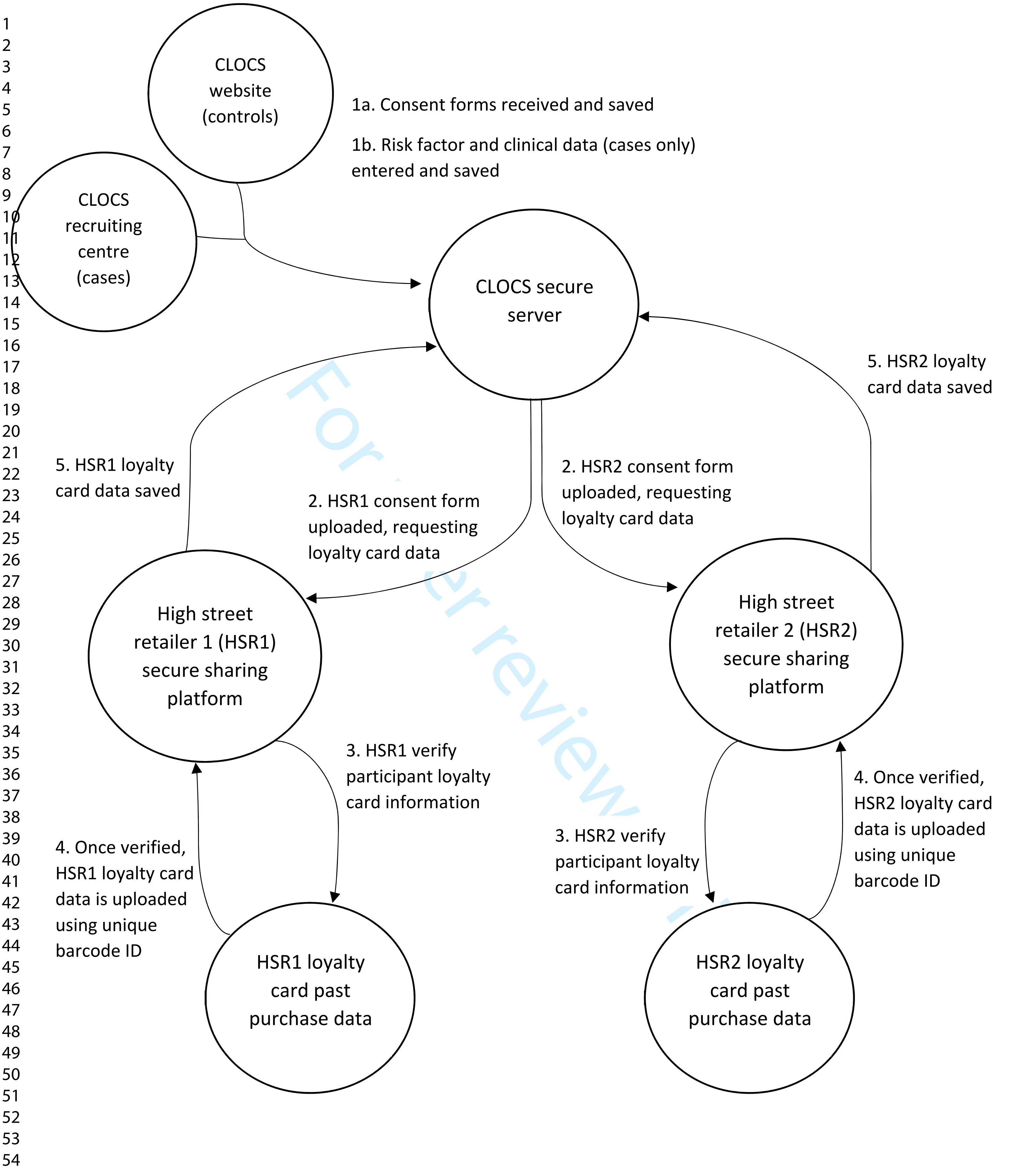

For peer review only - http://bmjopen.bmj.com/site/about/guidelines.xhtml

Supplementary Data

The following STROBE statement for case-control studies is the checklist CLOCS will use to report findings from the case-control study described in this protocol.

STROBE Statement—Checklist of items that should be included in reports of ***case-control studies***

	Item No	Recommendation
**Title and abstract**	1	(*a*) Indicate the study's design with a commonly used term in the title or the abstract
		(*b*) Provide in the abstract an informative and balanced summary of what was done and what was found
**Introduction**		
Background/rationale	2	Explain the scientific background and rationale for the investigation being reported
Objectives	3	State specific objectives, including any prespecified hypotheses
**Methods**		
Study design	4	Present key elements of study design early in the paper
Setting	5	Describe the setting, locations, and relevant dates, including periods of recruitment, exposure, follow-up, and data collection
Participants	6	(*a*) Give the eligibility criteria, and the sources and methods of case ascertainment and control selection. Give the rationale for the choice of cases and controls
		(*b*) For matched studies, give matching criteria and the number of controls per case
Variables	7	Clearly define all outcomes, exposures, predictors, potential confounders, and effect modifiers. Give diagnostic criteria, if applicable
Data sources/ measurement	8*	For each variable of interest, give sources of data and details of methods of assessment (measurement). Describe comparability of assessment methods if there is more than one group
Bias	9	Describe any efforts to address potential sources of bias
Study size	10	Explain how the study size was arrived at
Quantitative variables	11	Explain how quantitative variables were handled in the analyses. If applicable, describe which groupings were chosen and why
Statistical methods	12	(*a*) Describe all statistical methods, including those used to control for confounding
		(*b*) Describe any methods used to examine subgroups and interactions
		(*c*) Explain how missing data were addressed
		(*d*) If applicable, explain how matching of cases and controls was addressed
		(*e*) Describe any sensitivity analyses
**Results**		
Participants	13*	(a) Report numbers of individuals at each stage of study—eg numbers potentially eligible, examined for eligibility, confirmed eligible, included in the study, completing follow-up, and analysed
		(b) Give reasons for non-participation at each stage
		(c) Consider use of a flow diagram
Descriptive data	14*	(a) Give characteristics of study participants (eg demographic, clinical, social) and information on exposures and potential confounders
		(b) Indicate number of participants with missing data for each variable of interest
Outcome data	15*	Report numbers in each exposure category, or summary measures of exposure
Main results	16	(*a*) Give unadjusted estimates and, if applicable, confounder-adjusted estimates and their precision (eg, 95% confidence interval). Make clear which confounders were

For peer review only

	adjusted for and why they were included
	(*b*) Report category boundaries when continuous variables were categorized
	(*c*) If relevant, consider translating estimates of relative risk into absolute risk for a meaningful time period

For peer review only

For peer review only - http://bmjopen.bmj.com/site/about/guidelines.xhtml

Other analyses	17	Report other analyses done—eg analyses of subgroups and interactions, and sensitivity analyses
**Discussion**		
Key results	18	Summarise key results with reference to study objectives
Limitations	19	Discuss limitations of the study, taking into account sources of potential bias or imprecision. Discuss both direction and magnitude of any potential bias
Interpretation	20	Give a cautious overall interpretation of results considering objectives, limitations, multiplicity of analyses, results from similar studies, and other relevant evidence
Generalisability	21	Discuss the generalisability (external validity) of the study results
**Other information**		
Funding	22	Give the source of funding and the role of the funders for the present study and, if applicable, for the original study on which the present article is based

*Give information separately for cases and controls.

Note: An Explanation and Elaboration article discusses each checklist item and gives methodological background and published examples of transparent reporting. The STROBE checklist is best used in conjunction with this article (freely available on the Web sites of PLoS Medicine at http://www.plosmedicine.org/, Annals of Internal Medicine at http://www.annals.org/, and Epidemiology at http://www.epidem.com/). Information on the STROBE Initiative is available at http://www.strobe-statement.org.

For peer review only

For peer review only - http://bmjopen.bmj.com/site/about/guidelines.xhtml

BMJ Open

Cancer Loyalty Card Study (CLOCS): protocol for an observational case-control study focusing on the patient interval in ovarian cancer diagnosis

Journal:	*BMJ Open*
Manuscript ID	bmjopen-2020-037459.R2
Article Type:	Protocol
Date Submitted by the Author:	03-Jun-2020

Complete List of Authors:	Brewer, Hannah; Imperial College London, Surgery and Cancer<br>Hirst, Yasemin; University College London, Epidemiology and Public Health<br>Sundar, Sudha; University of Birmingham, Cancer Sciences<br>Chadeau-Hyam, Marc; Imperial College London, MRC Centre for Environment and Health; Imperial College London, Epidemiology and Biostatistics, School of Public Health<br>Flanagan, James; Imperial College London, Surgery and Cancer

**Primary Subject Heading**:	Oncology
Secondary Subject Heading:	Epidemiology, Public health
Keywords:	ONCOLOGY, Gynaecological oncology < GYNAECOLOGY, Epidemiology < ONCOLOGY, PUBLIC HEALTH

SCHOLARONE™ Manuscripts

I, the Submitting Author has the right to grant and does grant on behalf of all authors of the Work (as defined in the below author licence), an exclusive licence and/or a non-exclusive licence for contributions from authors who are: i) UK Crown employees; ii) where BMJ has agreed a CC-BY licence shall apply, and/or iii) in accordance with the terms applicable for US Federal Government officers or employees acting as part of their official duties; on a worldwide, perpetual, irrevocable, royalty-free basis to BMJ Publishing Group Ltd ("BMJ") its licensees and where the relevant Journal is co-owned by BMJ to the co-owners of the Journal, to publish the Work in this journal and any other BMJ products and to exploit all rights, as set out in our licence.

The Submitting Author accepts and understands that any supply made under these terms is made by BMJ to the Submitting Author unless you are acting as an employee on behalf of your employer or a postgraduate student of an affiliated institution which is paying any applicable article publishing charge ("APC") for Open Access articles. Where the Submitting Author wishes to make the Work available on an Open Access basis (and intends to pay the relevant APC), the terms of reuse of such Open Access shall be governed by a Creative Commons licence – details of these licences and which Creative Commons licence will apply to this Work are set out in our licence referred to above.

Other than as permitted in any relevant BMJ Author's Self Archiving Policies, I confirm this Work has not been accepted for publication elsewhere, is not being considered for publication elsewhere and does not duplicate material already published. I confirm all authors consent to publication of this Work and authorise the granting of this licence.

For peer review only

TITLE

Cancer Loyalty Card Study (CLOCS): protocol for an observational case-control study focusing on the patient interval in ovarian cancer diagnosis

Short Title

Cancer Loyalty Card Study (CLOCS): protocol for a case-control study

AUTHORS

Hannah R. Brewer[1], Yasemin Hirst[2], Sudha Sundar[3,4], Marc Chadeau-Hyam[5,6] and James M. Flanagan[1]

1. Department of Surgery and Cancer, Imperial College London, London, UK
2. Department of Behavioural Science and Health, University College London, London, UK
3. Pan-Birmingham Gynaecological Cancer Centre, City Hospital, Birmingham, UK.
4. Institute of Cancer and Genomic Sciences, University of Birmingham, Birmingham, UK.
5. MRC Centre for Environment and Health, Imperial College London, London, United Kingdom.
6. Department of Epidemiology and Biostatistics, School of Public Health, Imperial College London, London, United Kingdom.

Address for Correspondence:

Dr. James M. Flanagan

Division of Cancer, Department of Surgery and Cancer,

Faculty of Medicine, Imperial College London

4th Floor IRDB, Hammersmith Campus, Du Cane Road, London W12 0NN

Tel: 020 75941804; email: j.flanagan@imperial.ac.uk

For peer review only - http://bmjopen.bmj.com/site/about/guidelines.xhtml

For peer review only

ABSTRACT

Introduction

Ovarian Cancer is the eighth most common cancer in women worldwide, and about one in five women with ovarian cancer do not receive treatment, because they are too unwell by the time they are diagnosed. Symptoms of ovarian cancer are non-specific or can be associated with other common conditions, and women experiencing these symptoms have been shown to self-manage them using over-the-counter medication. Results from a recent proof of concept study suggest there may be an increase in the purchases of pain killers and indigestion medication 10-12 months before ovarian cancer diagnosis. We propose a case-control study, as part of a larger project called the Cancer Loyalty Card Study (CLOCS), to investigate whether a significant change in medication purchases could be an indication for early signs of ovarian cancer, using data already collected through store loyalty cards.

Methods and Analysis

Using a retrospective case-control design, we aim to recruit 500 women diagnosed with ovarian cancer (cases) and 500 women without ovarian cancer (controls) in the United Kingdom who hold a loyalty card with at least one participating high street retailer. We will use pre-existing loyalty card data to compare past purchase patterns of cases with those of controls. In order to assess ovarian cancer risk in participants and their purchase patterns, we will collect information from participants on ovarian cancer risk factors and clinical data including symptoms experienced before diagnosis from recruited women with ovarian cancer.

Ethics and dissemination

CLOCS was reviewed and approved by the North West - Greater Manchester South Research Ethics Committee (19/NW/0427). Study outcomes will be disseminated through academic publications, the study website, social media, and a report to the research sites that support the study once results are published.

Study Registration Numbers

CLOCS is registered with ISRCTN (14897082), NIHR portfolio (CPMS 43323), and clinicaltrials.gov (NCT03994653).

Keywords:

Ovarian cancer, early detection, purchase behaviour, observational case-control study, commercial data

For peer review only - http://bmjopen.bmj.com/site/about/guidelines.xhtml

Strengths and limitations of this study

- This study is a novel approach to investigate whether routinely collected commercial purchase data can be used to reduce delays in ovarian cancer diagnosis.
- Ovarian cancer advocates have been involved in the conception, design and conduct of the study.
- A key strength of the study is the detailed information available for purchases recorded for up to 7 years prior to enrolment.
- A key limitation is that loyalty card holders may not use their cards for every purchase, may shop at other stores not being accessed in this study and may be purchasing items for other family members.
- In general, due to the retrospective nature of case-control studies, there is potential for the misreporting of exposures in the risk factor questionnaire by cases and controls (recall bias).

INTRODUCTION

Approximately 7,200 new cases of ovarian cancer are diagnosed each year in the UK, and with over 4,200 women dying from the disease each year, it is a particularly lethal form of cancer[1]. Diagnosing ovarian cancer in primary and secondary care settings among symptomatic patients is a challenge due to their low positive predictive value of symptoms and symptom presentation at various stages of the ovarian cancer prognosis[2, 3]. Symptoms, such as increased abdominal size, pelvic pain, abdominal pain and bloating, feeling full quickly and difficulty eating have been identified as high risk symptoms but equally it has been reported that the absence of a symptom does not help ruling out ovarian cancer[4, 5]. For patients whose symptoms do not warrant an immediate referral earlier presentation in primary care is crucial to reduce delays in diagnosis and subsequent treatment.

However, symptom recognition and help-seeking in primary care are also not without challenges. While in some cases patients may present very quickly, for some patients it could take up to 12 months before they seek any medical help[6]. It has been shown that greater awareness of symptoms alone does not facilitate help seeking for ovarian cancer because symptoms are often perceived as vague or trivial[7, 8]. The major reasons for not presenting to the primary care are often highlighted as “not wanting to waste the GP’s time”[9],

For peer review only - http://bmjopen.bmj.com/site/about/guidelines.xhtml

normalisation of these symptoms[10], and few women believing that their symptom(s) might be a sign of cancer[11]. Women might choose to self-manage such symptoms by seeking advice from sources other than a primary care physician, e.g. pharmacists, family, internet, and using over-the-counter medication[12] which may also have been moderated by other factors such as access to healthcare, sociodemographic factors and other competing priorities, e.g. work and care responsibilities[7-11].

There are currently no organised screening programmes offered to women that could help detect ovarian cancer at an earlier stage, and there is little evidence for effective interventions aiming to improve symptom recognition and help-seeking.

Therefore, novel methods are needed to improve earlier detection of low-volume disease or earlier stage to improve survival[13]. A recent proof of concept study investigated whether purchase information collected through the high street retailers' loyalty cards (e.g. store points cards) could be used to identify women's self-care behaviours prior to presenting to health care professionals. This study included data from a major high street retailer's loyalty card of women who have previously been diagnosed with ovarian cancer and consented their past purchase data to be provided to the researchers. The study suggested that there may be an increase in the purchases of pain killers and indigestion tablets up to 10-12 months before diagnosis[14]. This proof-of-concept study provided a new model of investigation for symptom recognition and help-seeking among ovarian cancer patients. It highlighted potential strengths and limitations in its methodology for future research. Building on these results, self-care behaviours of ovarian cancer patients will be further investigated with a case-control study within a larger project called Cancer Loyalty Card Study (CLOCS). CLOCS hypothesises that a significant change in purchasing behaviour, primarily an increase in self-medication or self-care behaviours to treat symptoms prior to presentation in primary care (see Figure 1), could be an indication for early signs of ovarian cancer.

Study aims

The primary aim of this case-control study is to investigate pre-diagnosis purchasing behaviours of ovarian cancer patients (cases) with women without a previous ovarian cancer diagnosis (controls) using data collected through two major high street retailers' loyalty card schemes. We will investigate purchase behaviour of items that are suspected to treat symptoms of ovarian cancer (e.g. painkillers, indigestion tablets) as well as a change in frequency or quantity of other purchased items. The secondary aim of CLOCS will be to

For peer review only - http://bmjopen.bmj.com/site/about/guidelines.xhtml

investigate whether or not a purchase threshold as an "alert" about potential ovarian cancer symptoms in individuals can be possible using loyalty card data and determining the predictive utility of purchasing behaviours in the early detection of ovarian cancer based on ovarian cancer risk. This would be important to inform if future interventions would be possible using loyalty card data. Future interventions could include an alert sent to individuals to visit a GP and a potential public health message about those purchase thresholds and self-treatment of ovarian cancer symptoms.

METHODS AND ANALYSIS

Study design and setting

CLOCS will use a retrospective case-control design to compare past purchase patterns of women with and without ovarian cancer using already existing data gathered by two major high street retailers, one of which is a health and beauty retailer and the other a groceries and general merchandise retailer. Furthermore, we will collect data on risk factors for ovarian cancer, symptoms experienced before diagnosis and clinical data from ovarian cancer patients using self-reported questionnaires.

CLOCS will be recruiting across the United Kingdom from at least 40 National Health Service (NHS) Trusts and Hospitals and recruit healthy volunteers using online recruitment.

Study timeline

The CLOCS case-control study will take place from 1st November 2019 until 1st February 2022.

Participants

Inclusion/Exclusion Criteria

Cases

Women, at least 18 years old, diagnosed and living with any type of ovarian cancer (including all subtypes of epithelial ovarian cancer high-grade serous, low-grade serous, endometrioid, clear cell, mucinous, borderline, and other subtypes) within the previous 2 years from the recruitment date who own at least one participating high street retailer loyalty card are eligible to join CLOCS case-control study as cases.

There are no further clinical criteria for patients to be excluded from this study. Patients' usual care will continue to be provided by the health care professionals, and CLOCS

For peer review only - http://bmjopen.bmj.com/site/about/guidelines.xhtml

participation does not exclude patients from participating in other clinical or non-clinical studies.

Controls

Women, at least 18 years old, who have not been diagnosed with ovarian cancer and own at least one of the loyalty cards from the participating retailers are eligible to join the CLOCS as controls. We are not excluding women with previous cancer diagnosis, or women who may have had their ovaries removed for reasons other than cancer; however, these will be considered when cases are matched with controls.

Recruitment

The CLOCS case-control study recruitment process is outlined in figure 2 and described further detail below.

Cases

Eligible ovarian cancer patients will be recruited by members of their healthcare teams at approximately 40 NHS Trusts and Hospitals across the United Kingdom that have agreed to approach patients at clinics. These healthcare teams are registered as research sites and will have a principal investigator (PI) leading the recruitment to CLOCS. The PI and the research nurses will facilitate recruitment by identifying patients when they think it is suitable to approach patients based on patients' overall well-being at a suitable time (e.g. at waiting areas, during chemotherapy sessions), then will provide patients with the study information to consider participation. Patients will be given a paper-based participant pack that includes study consent form including consents for providing access to data collected by the retailers, self-report questionnaire, and a clinical questionnaire to be completed by the clinical staff facilitating the recruitment. If they wish to participate, patients can complete all the study documents at the recruitment site and post it back to the research team using the free-post envelope provided in the study pack, or they are also given the option to consider participation at home provided that the clinical questionnaire has been completed by a member their clinical team. The research sites will not need to record any data from the participants. The data collection from the cases will be a one-off process and no further contact with the patient is required for the study.

Controls

The control participants to the study will be recruited through the study website (www.clocsproject.org.uk) where data collection will be safeguarded using an encrypted web form that includes study consent and the self-report questionnaire. The website has been

For peer review only - http://bmjopen.bmj.com/site/about/guidelines.xhtml

developed and maintained by NDP limited. The questionnaire for women without ovarian cancer will be confined to risk factors. The online participation will take up to 15 minutes and data will be transferred immediately to the secure CLOCS server (see figure 3). The website approach was adopted as one of the outcomes of the proof-of-concept study. It aims to provide a transparent environment where potential participants can make informed decisions about participation in CLOCS, and the data risks are minimised using encryption. We have carefully designed the website to inform the general public about how we will process their data in CLOCS, how they can withdraw from the study if they wish to do so and use it as a platform to provide feedback as we progress in the larger CLOCS project. The research team will use several methods to recruit control participants to the study which will include a press release carried out by the project funders and the Imperial College London public engagement team, dissemination of study flyers and leaflets to patients in participating NHS recruitment clinics, and social media marketing techniques on Twitter and Facebook using paid advertisement and unpaid methods. The application of the social media recruitment methods will allow researchers to reach a larger cohort in the UK. The application of both paper-based and online recruitment methods will be carried out throughout CLOCS and will be recorded. The efficacy of these methods will be reported in peer-reviewed journals to inform online research recruitment to future case-control studies.

Data sources and variables included

Loyalty Card Data (For cases and controls)

CLOCS participants will hold a loyalty card at one or both participating retailers, including two health, beauty and/or grocery retailers that can be accessed by the UK population within all precincts allowing the study to assess purchases of painkillers and indigestion medication as well as eating habits. The study will not include prescription-based pharmacy data. Once participants consent for the CLOCS team to request their loyalty card data from the retailers on their behalf, the study will include individuals' past purchase data from up to six years prior to the date of study consent. The purchase data will include each individual purchase, date of purchase, the location (i.e. store postcode), and the product categorisation that is provided by the retailers. There will be no additional individual information recorded by the study team from this dataset and no additional individual information will be requested from the retailers. Please note that at this stage of the study, we will not be naming the high street retailers in publications and in our direct communications, but they are named to the potential participants when they agree to take part.

For peer review only - http://bmjopen.bmj.com/site/about/guidelines.xhtml

Ovarian Cancer Risk Questionnaire (For Cases and Controls)

In order to stratify the purchase differences between cases and controls based on ovarian cancer risk, we designed a questionnaire on ovarian cancer risk factors that will allow us to determine individuals' ovarian cancer risk. Both cases and control participants will be asked to complete a short questionnaire about ethnicity, marital status, and the following well-established ovarian cancer risk factors: body mass index, age at menarche, menopausal status, age at menopause, parity, breastfeeding, hysterectomy, tubal ligation, cancer history, endometriosis, aspirin use, oral contraceptive use, hormone replacement therapy (HRT), family history of ovarian and breast cancers, vaping, and cigarette smoking[15]. These factors will be used to assess ovarian cancer risk among participants to distinguish high-risk and low-risk individuals and subsequently observe patterns in purchase history stratified by risk levels.

The questionnaire also includes questions about the symptoms experienced (if any) and number of visits to the general practitioner in the year leading up to cancer referral or diagnosis which were adopted from the proof-of-concept study[14].

The final section of the questionnaire contains questions about which food and pharmacy stores participants shop at regularly, at which stores they own a loyalty card, and frequency of use of those loyalty cards.

Clinical Questionnaire (Cases only)

Women with an ovarian cancer diagnosis will also have a clinical questionnaire completed by their healthcare team. This will provide the objective timeline and pathway for diagnosis (e.g. GP referral, emergency presentation, other), details on the type (high-grade serous, low-grade serous, endometrioid, clear cell, mucinous, borderline, and other), stage (I, II, III, or IV), grade of ovarian cancer at diagnosis (grade 1 or well differentiated, 2 or moderately differentiated, 3 or poorly differentiated/undifferentiated), surgical outcome if any (optimal debulking, sub-optimal debulking, or no surgery), and BRCA status if the participant has been tested (BRCA1 positive, BRCA2 positive, both negative, awaiting result). We will use this information in risk assessment and to stratify the loyalty card data of cases and controls based on the time of diagnosis, and observe purchase history stratified by type, stage, and grade of ovarian cancer diagnoses.

Data collection processes (see figure 3)

In the next section we will describe, how we will be collecting and processing data in the CLOCS case-control study.

For peer review only

1) Data Acquisition

Upon choosing to participate, both cases and controls will need to complete a consent form to participate in CLOCS and return it to the CLOCS research team either by post (cases only) or the CLOCS website (controls only). The CLOCS consent form includes two sections. The first section details consent to take part in CLOCS, and the second section is for participants to consent to the CLOCS research team to request their data from high street retailers on their behalf. The consents for loyalty cards include the exact name of the individual and the exact membership number as written on their card. All individual consents including scanned copies of paper consents will be saved in the secure and encrypted server based in the "Secure Enclave" at Imperial College London. Participant loyalty card details will be entered in to a comma-separated values (CSV) file and encrypted for secure data request from retailers using a secure file transfer protocol (SFTP).

2) Data Extraction from the high street retailers

The CLOCS team will send the CSV file that contains a unique study ID, name of the individual and the exact loyalty card number specific to the retailer using the secure file transfer. The respective high street retailer will validate the participant information from the consent form with their loyalty card data. If loyalty card details cannot be validated, the consents and any information from those individuals who have not consented to be re-contacted will be excluded from the study and deleted immediately. High street retailers will then generate a dataset for all consenting individuals and will share the past purchase data with the CLOCS team using the SFTP. Depending on the high street retailer's records and when the participant obtained the loyalty card, up to 7 years of participants' past purchasing data prior to the date of recruitment will be shared.

3) Data Merge

The CLOCS team will receive the encrypted participant purchase history from the high street retailers through the SFTP, save, and store all data to a secure and encrypted server based in the Secure Enclave at Imperial College London, described in *Data management*. All data will be pseudonymised and analysed on the secure server.

4) Data Deletion

For peer review only - http://bmjopen.bmj.com/site/about/guidelines.xhtml

The information relating to the participants' consents for providing past purchase data will be retained in line with each high street retailers' data retention policy. Ten years after the study is complete, all paper-based documents will be shredded and destroyed in accordance with Imperial College London's data deletion and retention processes. Electronically held identifiable data including individual consent and contact details will be kept for 10 years after the study is complete and will be deleted from the secure server in accordance with Imperial College London's data deletion and retention processes. Past purchase data will be kept until the results are published or five years from the date of participation in the study, whichever comes sooner, before it will be anonymised and the link to all identifiable data will be deleted. During the lifetime of the study, participants are given the option to withdraw consent which will mean that all data associated with that individual will be deleted and records will be removed from CLOCS.

Sample size and statistical power

Based on the modelled data from the pilot study[14], we hypothesise that we could detect a difference in purchase proportions of 8.3% at 3 months prior to diagnosis, 6.3% at 6 months, 4.3% at 9 months and 2.3% at 12 months prior to diagnosis between cases and controls. Using the two-sample test for proportions the minimum group size to detect these differences at 80% power and α=0.05 would be 125 (-3 months), 185 (-6 months), 328 (-9 months) and 969 (-12 months). We modelled the power versus time prior to diagnosis and chose 10 months as our target to retain sufficient power for larger effect sizes at earlier time points. Therefore, in the final analysis we will have >80% power at α=0.05 to detect an increase in proportion of purchases in cases (>4.7%) vs controls (1.4%) at 10 months prior to diagnosis, based on the modelled data, with a minimum n=431 cases. We aim to recruit 500 participants to account for 10-15% of very low frequency card users. We will compare proportions using Fishers Exact Test comparing target (items suspected to be used to treat ovarian cancer symptoms) and non-target (all other items) purchases in cases at each time point prior to diagnosis compared with the average monthly proportions in the controls. Target items for analysis will include but will not be limited to medications for pain, indigestion, and bloating relief (see table 1 in Appendix A of the supplementary material for a list of example purchases). We will report proportions, 95% confidence intervals and *P*-values.

Statistical Methods

For peer review only - http://bmjopen.bmj.com/site/about/guidelines.xhtml

We will use descriptive statistics to provide details on the characteristics of the cases and controls by sociodemographic (e.g. age, ethnicity) and by risk factors (e.g. parity, oral contraceptive use, etc.). We will report feasibility factors such as number of people who had both, or one loyalty card, and number of years of data included in the study.

Cases and controls will be matched 1:1 by age (5-year age groups) and ethnicity. Ethnicity will be matched based on the guidance from the Office for National Statistics[16]. We will aim to define the time by which cases and controls become statistically significantly different in their purchase behaviours using a multivariable, conditional logistic regression model with ovarian cancer as the outcome and "purchase proportion" at different time points to diagnosis as the exposure, adjusting for stage and histology and other potentially confounding variables collected from the risk factor questionnaire (including body mass index, age at menarche, menopausal status, age at menopause, parity, breastfeeding, hysterectomy, tubal ligation, cancer history, endometriosis, aspirin use, oral contraceptive use, hormone replacement therapy (HRT), family history of ovarian and breast cancers, vaping, and cigarette smoking[15]). We will compare the proportions of purchases in cases to controls, stratified by risk quintiles defined by the risk model (estimated n=86 per quintile) and by histology (with expected proportions of high-grade serous, mucinous cases, endometrioid, clear cell, and low-grade serous cases). Analyses will be performed for different types of medications separately and combined (e.g. a comparison of case-control purchase proportion of painkillers alone and indigestion medications alone, and then of painkillers and indigestion medications combined).

Established risk factors have been reported to modestly discriminate between cases and controls (AUC=0.66)[17], and polygenic risk scores only slightly improves these performances. This model has however been proposed to improve epithelial ovarian cancer detection with risk stratified screening[18]. We will estimate the predictive values of this "early diagnosis" test for each stratum.

We will conduct exploratory analyses investigating other methods leveraging the auto correlation in the longitudinal data. Specifically, we will explore the evolutions of individual frequency of purchases and purchase trajectories, to identify purchase patterns (e.g. increasing purchases before diagnosis, purchase peaks, etc.), that are indicative/predictive of the disease onset and of the time-to-onset.

All analyses will be done using the statistical software R.

Interim Analysis

For peer review only - http://bmjopen.bmj.com/site/about/guidelines.xhtml

An interim analysis will be conducted after six months of CLOCS recruitment to provide an initial exploratory analysis of the data and assess the demographics of CLOCS participants. This will help determine whether recruitment will need to target specific demographics (e.g. age groups) in order to ensure there is a representative distribution of ages and ethnicities for the matched analysis.

Study risks and sources of bias

There are no clear risks for participants in the CLOCS. However, it may be emotional or distressing for patients to think about cancer and the first time they noticed some changes in their body (i.e. symptoms). The CLOCS team intended to design the survey questions in the most sensible and sensitive way to ensure that there are no negative effects of this study on patients' well-being. All participants are advised to see a medical professional in the information sheet if they are worried about a symptom or sign that they might have been experiencing.

For many loyalty card holders, purchases will be for themselves and other individuals in their household. The CLOCS team will not be able to quantify what purchases are consumed by the participant and by other members of the household, but the team will be able to make assumptions to adjust accordingly.

The CLOCS team acknowledges that not all participants will have used their loyalty card with every purchase for various reasons (e.g. card not with them at time of purchase, in a hurry, etc.) and that participants will have made purchases at other shops not recorded with their loyalty cards observed in this study. For this reason, the risk factor questionnaire asks participants to indicate how often they use their loyalty card when shopping, at which stores they shop most regularly and own a loyalty card, so that these can be taken account at the analysis stage.

It is possible that CLOCS participants could have other comorbidities, and their over-the-counter purchases might be used to treat symptoms from those instead of symptoms of ovarian cancer. If women have been diagnosed with comorbidities (e.g. asthma, diabetes, osteoporosis, depression, etc. [19]), we would assume that they might already be receiving prescribed medication to treat these from their healthcare providers. If they are using prescribed medication, then loyalty card data does not include information about prescriptions. Common comorbidities that are not yet diagnosed or that do not require prescription medication are expected to be present in similar frequencies among CLOCS participants with and without ovarian cancer. The risk factor questionnaire will address some of these comorbidities, which will allow us to adjust for them in the analysis.

For peer review only - http://bmjopen.bmj.com/site/about/guidelines.xhtml

It is also possible that ovarian cancer patients may have already been seen by a health care professional for their symptoms and may have been prescribed medication for reasons other than ovarian cancer, e.g. urinary tract infection, irritable bowel syndrome, prior to being referred for suspected ovarian cancer. However, access to prescribed medication information would require access to primary care records and not included in this study. It would also mean that patients have already sought help for their symptoms and the patient interval phase for symptom appraisal before help-seeking would have been finalised. We will control for this by adjusting for the self-reported number of visits to the GP leading up to the diagnosis. This would also be discussed as a potential limitation, and we will be cautious in our evaluations of the results if we observe a reduction in risk related purchases close to the date of diagnosis.

CLOCS limits participation to women who own a loyalty card from at least one of the participating retailers. Approximately 73% of women aged 50-80 years in the UK have a loyalty card to one of the participating high street retailers, and therefore the cardholders in CLOCS should be representative. Nevertheless, there will be selection bias against those who do not hold a loyalty card.

In any retrospective case-control study, there is the potential for recall bias. Participants with ovarian cancer could report experiencing more symptoms more frequently than may have occurred, but for those who report they self-managed these symptoms, the CLOCS team will be able to observe loyalty card purchase data to validate the amount and frequency of self-medication prior to diagnosis.

Data management

The CLOCS research team will act as *data processors* when requesting data from high street retailers and the high street retailers would be *data controllers*. CLOCS will become the data controller for the self-reported questionnaire data and the clinical questionnaire data. The consenting participants will be *data owners* and will have the right to withdraw their consent from the study at any time without giving any reason to the study team.

Only the CLOCS research team will have access to the paper-based consent forms which will be kept in a locked cupboard and server in a locked room and secure building that requires security badge access to enter. The data will be analysed in a secure environment with access limited to the research team. Participants will be given a unique study ID barcode which means all datasets will be pseudonymised.

All data will be stored and processed in Imperial College's ISO27001 certified secure environment – the "Secure Enclave". This is a fully managed infrastructure and secure

For peer review only - http://bmjopen.bmj.com/site/about/guidelines.xhtml

environment providing high availability, resilience and business continuity through multiple servers, back-ups and disaster recovery measures. A robust data security model has been designed to protect sensitive personal and medical data from the potential risk of unauthorised access or distribution. No one other than the research team will have access to the research dataset.

The CLOCS website is compliant with the high level of data security required by Imperial College London and General Data Protection Regulation (GDPR). The data collected through the website only resides in the Secure Enclave and not in the browser or website. It also uses Public Key Encryption, which encrypts data from the site that can only be unlocked once within the enclave. These requirements are equalled by support for the user to complete the online survey, by using content and interfaces that are easy to use, especially on mobile phones.

We have strict data processing agreements in place with our commercial partners to ensure that data is protected at all times. Commercial partners will not have access to the study data and will not financially benefit from this project.

Patient and public involvement

Ovarian cancer patients and members of the public have been involved in the development of the CLOCS from the beginning, participating in the proof-of-concept study that assessed the general public's acceptability of the use of commercial and health data linkages for cancer symptom surveillance[14]. Two ovarian cancer patient advocates who participated in the pilot study have taken part in research meetings developing this case-control study and reviewed study documentation. They have been and will continue to be involved in ongoing development of CLOCS methods and dissemination of the research results through social media, media outlets and patient groups. The questionnaire for women without ovarian cancer has also been reviewed and approved by members of the public.

We also use our website for providing information to the patients and public. For instance, all research goals, how we will be analysing data, confidentiality and data security are all detailed for the lay population. There is an animation hosted on our website that is aiming to explain the study in simpler words. All materials are reviewed and approved by patient advocates. We carry out annual research meetings with stakeholders including research nurses, patients, academic collaborators, retailers, funders and public to provide updates and receive feedback on this project.

For peer review only - http://bmjopen.bmj.com/site/about/guidelines.xhtml

ETHICS AND DISSEMINATION

CLOCS was reviewed and approved by the North West - Greater Manchester South Research Ethics Committee (19/NW/0427). The study is registered as an NHIR portfolio study (Ref: CPMS 43323), with the ISRCTN (14897082), and is registered on clinicaltrials.gov (NCT03994653). This paper describes the ethics approved protocol dated 8 August 2019 (version 2).

We will disseminate the outcomes of the study using academic publications, the study website (clocsproject.org.uk), social media (@CLOCS_Imperial), and send a report to the research sites that supported the study once the results are published. We will aim to publish the results in a peer-reviewed journal in accordance with the STROBE statement for case-control studies[20]. The STROBE statement we intend to use when reporting the results from this case-control study is listed in Appendix B in the supplementary material. We will also use the study website to inform the public about the study outcomes.

Data Sharing Policy

All access to the CLOCS data collected from the retailers will be restricted and will not be shared with other researchers due to the data sharing agreements with the retailers. However, we do encourage other researchers to contact us for collaborations. If they are interested, they are given the opportunity to review the metadata by directly contacting us and submit a research proposal where the data analyses will be carried out by a member of the CLOCS team. All proposals will be reviewed case by case by the PI and the rest of the research team.

For peer review only

For peer review only - http://bmjopen.bmj.com/site/about/guidelines.xhtml

Authors' contributions:

JMF and YH conceived the study. HRB wrote the first draft of the manuscript. YH, JMF, SS and MC-H commented and reviewed the final draft of the manuscript. All authors agree on the final version of the protocol.

Funding statement:

Cancer Loyalty Card Study (CLOCS) is funded by Cancer Research UK (CRUK) Early Detection Project Grant (C38463/A26726). YH and HRB are funded by CRUK.

Competing interests statement

The authors declare no competing interests.

Acknowledgements

We thank NDP Studio Limited for their support and development of the study website (www.clocsproject.org.uk). We thank our patient and public representatives for reviewing the study materials and providing guidance on study acceptability from the conception of CLOCS to data collection. The authors acknowledge infrastructure and recruitment support from the Imperial Experimental Cancer Medicine Centre, Cancer Research UK Imperial Centre, the National Institute for Health Research Imperial Biomedical Research Centre, and the Ovarian Cancer Action Research Centre. We thank Boots UK employees who facilitated the project for their support and guidance during the development of this project. We thank Eric Johnson for guidance on data security and GDPR.

Figure Legends

Figure 1: Pathway to diagnosis highlighting the patient interval that CLOCS will investigate.

Figure 2: CLOCS recruitment process.

Figure 3: CLOCS data flowchart.

For peer review only

REFERENCES

1. Cancer Research UK. Ovarian cancer statistics. https://www.cancerresearchuk.org/health-professional/cancer-statistics/statistics-by-cancer-type/ovarian-cancer. Accessed 9 Apr 2019.

2. Funston G, Van Melle M, Baun M, Jensen H, Helsper C, Emery J, et al. Variation in the initial assessment and investigation for ovarian cancer in symptomatic women: A systematic review of international guidelines. BMC Cancer. 2019.

3. National Institute for Health and Care Excellence. Ovarian cancer: recognition and initial management. Clinical guideline [CG122]. 2011.

4. Ebell M, Culp M, Radke T. A Systematic Review of Symptoms for the Diagnosis of Ovarian Cancer. American Journal of Preventive Medicine. 2016;50:384–94. doi:10.1016/j.amepre.2015.09.023.

5. Goff B. Symptoms associated with ovarian cancer. Clin Obstet Gynecol. 2012;55:36–42.

6. Hamilton W, Peters TJ, Bankhead C, Sharp D. Risk of ovarian cancer in women with symptoms in primary care: Population based case-control study. BMJ. 2009;339:616.

7. Brain K, Smits S, Simon A, Forbes L, Roberts C, Robbé I, et al. Ovarian cancer symptom awareness and anticipated delayed presentation in a population sample. BMC Cancer. 2014.

8. Low E, Waller J, Menon U, Jones A, Reid F, Simon A. Ovarian cancer symptom awareness and anticipated time to help-seeking for symptoms among UK women. J Fam Plan Reprod Heal Care. 2013.

9. ICBP. The international cancer benchmarking partnership evidence for policy and practice October 2016. 2016.

10. Low E, Whitaker K, Simon A, Sekhon M, Waller J. Women's interpretation of and responses to potential gynaecological cancer symptoms: A qualitative interview study. BMJ Open. 2015;5.

11. Whitaker K, Winstanley K, Macleod U, Scott S, Wardle J. Low cancer suspicion following experience of a cancer "warning sign." Eur J Cancer. 2015;51:2473–9.

12. Jayde V, Boughton M. The diagnostic journey of ovarian cancer: A review of the literature and suggestions for practice. Contemporary Nurse. 2012.

13. Gilbert L, Basso O, Sampalis J, Karp I, Martins C, Feng J, et al. Assessment of

For peer review only - http://bmjopen.bmj.com/site/about/guidelines.xhtml

symptomatic women for early diagnosis of ovarian cancer: results from the prospective DOvE pilot project. Lancet Oncol. 2012;13:285–91. doi:10.1016/S1470-2045(11)70333-3.

14. Flanagan J, Skrobanski H, Shi X, Hirst Y. Self-Care Behaviors of Ovarian Cancer Patients Before Their Diagnosis: Proof-of-Concept Study. JMIR Cancer. 2019;5:e10447. doi:10.2196/CANCER.10447.

15. Reid B, Permuth J, Sellers T. Epidemiology of ovarian cancer: a review. Cancer Biol Med. 2017;14:9–32. doi:10.20892/j.issn.2095-3941.2016.0084.

16. Office for National Statistics. Ethnic group, national identity and religion, Measuring equality: A guide for the collection and classification of ethnic group, national identity and religion data in the UK. 2016. https://www.ons.gov.uk/methodology/classificationsandstandards/measuringequality/ethnicgroupnationalidentityandreligion#toc.

17. Clyde M, Palmieri Weber R, Iversen E, Poole M, Doherty J, Goodman M, et al. Risk Prediction for Epithelial Ovarian Cancer in 11 United States-Based Case-Control Studies: Incorporation of Epidemiologic Risk Factors and 17 Confirmed Genetic Loci. Am J Epidemiol. 2016;184:555–69.

18. Meisel SF, Side L, Fraser L, Gessler S, Wardle J, Lanceley A. Population-based, risk-stratified genetic testing for ovarian cancer risk: A focus group study. Public Health Genomics. 2013;16:184–91.

19. Minlikeeva AN, Freudenheim JL, Eng KH, Cannioto RA, Friel G, Szender JB, et al. History of comorbidities and survival of ovarian cancer patients, results from the ovarian cancer association consortium. Cancer Epidemiol Biomarkers Prev. 2017;26:1470–3.

20. von Elm E, Altman DG, Egger M, Pocock SJ, Gøtzsche PC, Vandenbroucke JP. The Strengthening the Reporting of Observational Studies in Epidemiology (STROBE) statement: guidelines for reporting observational studies. Lancet. 2007.

For peer review only - http://bmjopen.bmj.com/site/about/guidelines.xhtml

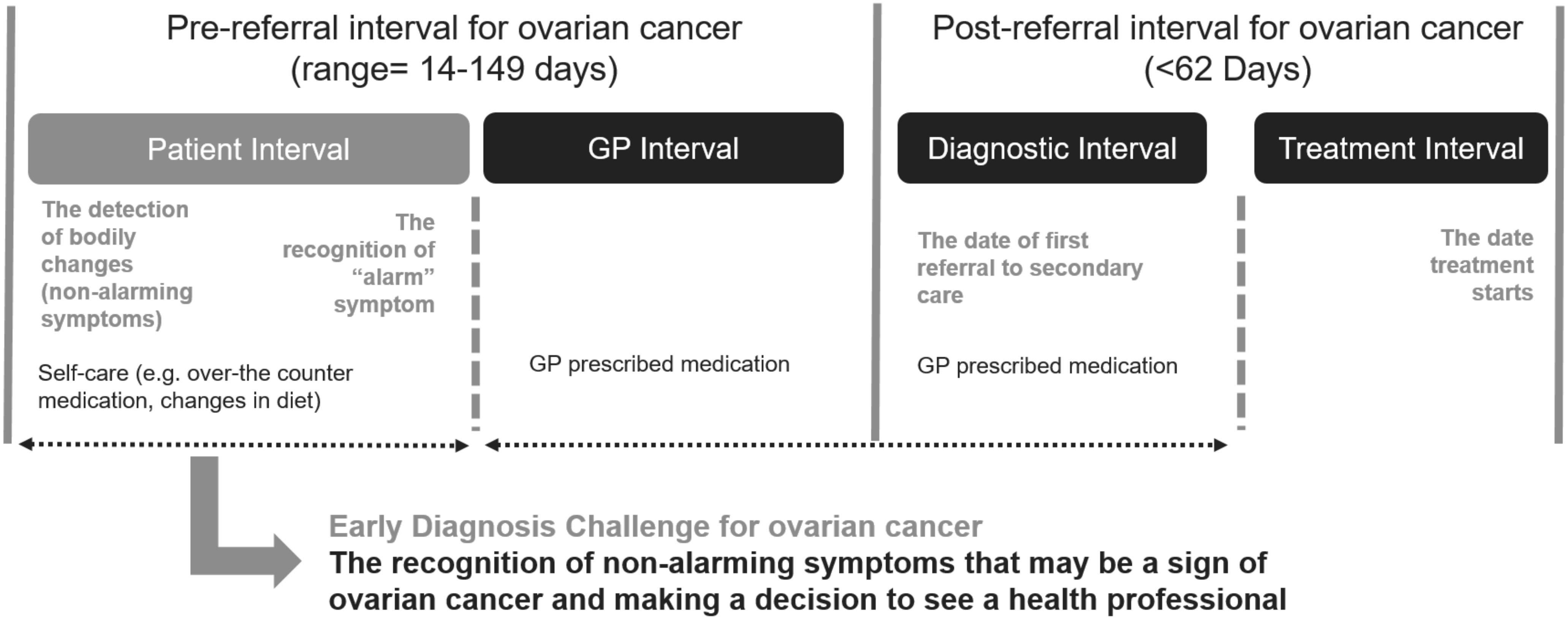
Pre-referral interval for ovarian cancer
(range= 14-149 days)
Post-referral interval for ovarian cancer
(<62 Days)
Patient Interval
GP Interval
Diagnostic Interval
Treatment Interval
The detection of bodily changes (non-alarming symptoms)
The recognition of "alarm" symptom
The date of first referral to secondary care
The date treatment starts
Self-care (e.g. over-the counter medication, changes in diet)
GP prescribed medication
GP prescribed medication
Early Diagnosis Challenge for ovarian cancer
The recognition of non-alarming symptoms that may be a sign of ovarian cancer and making a decision to see a health professional

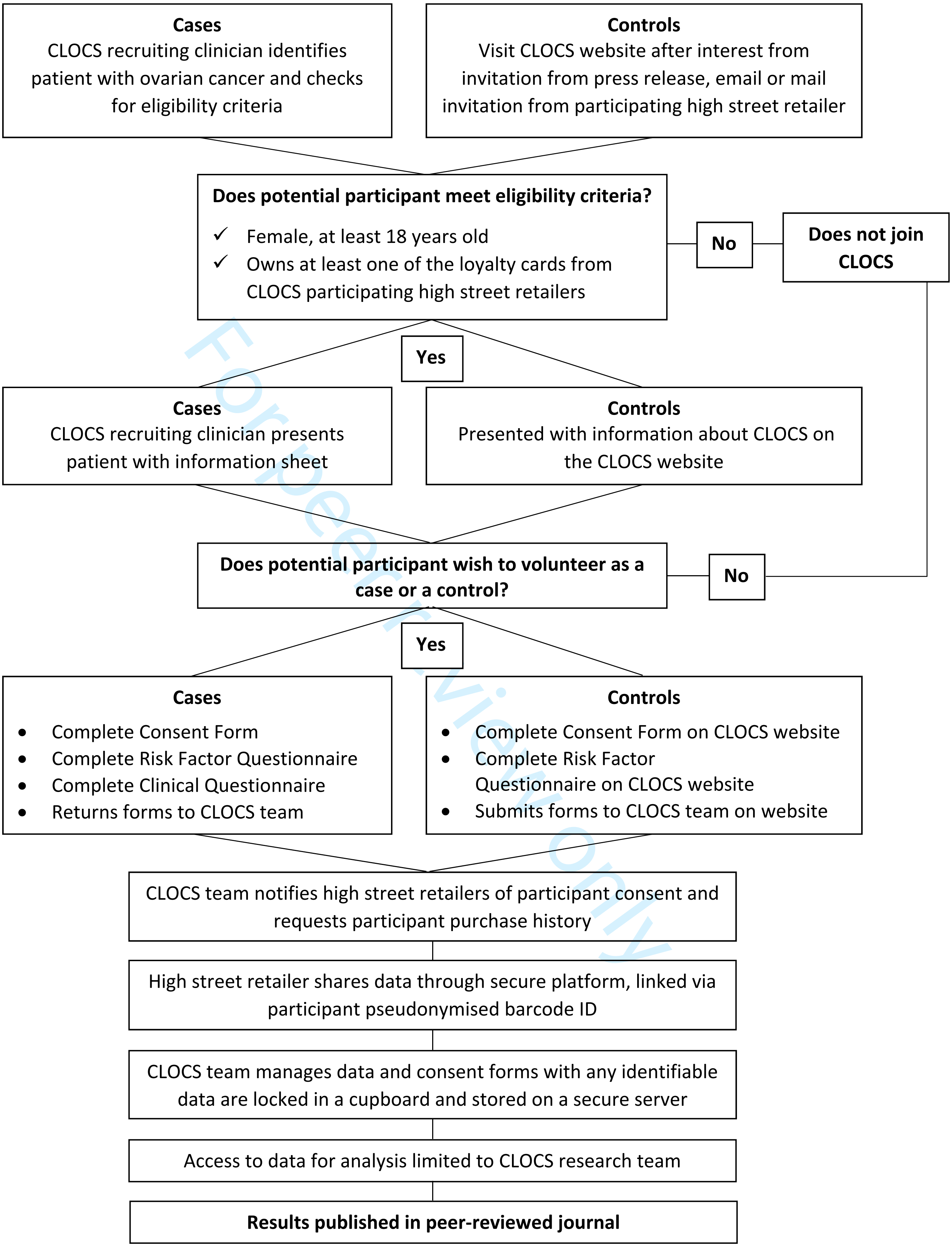

For peer review only

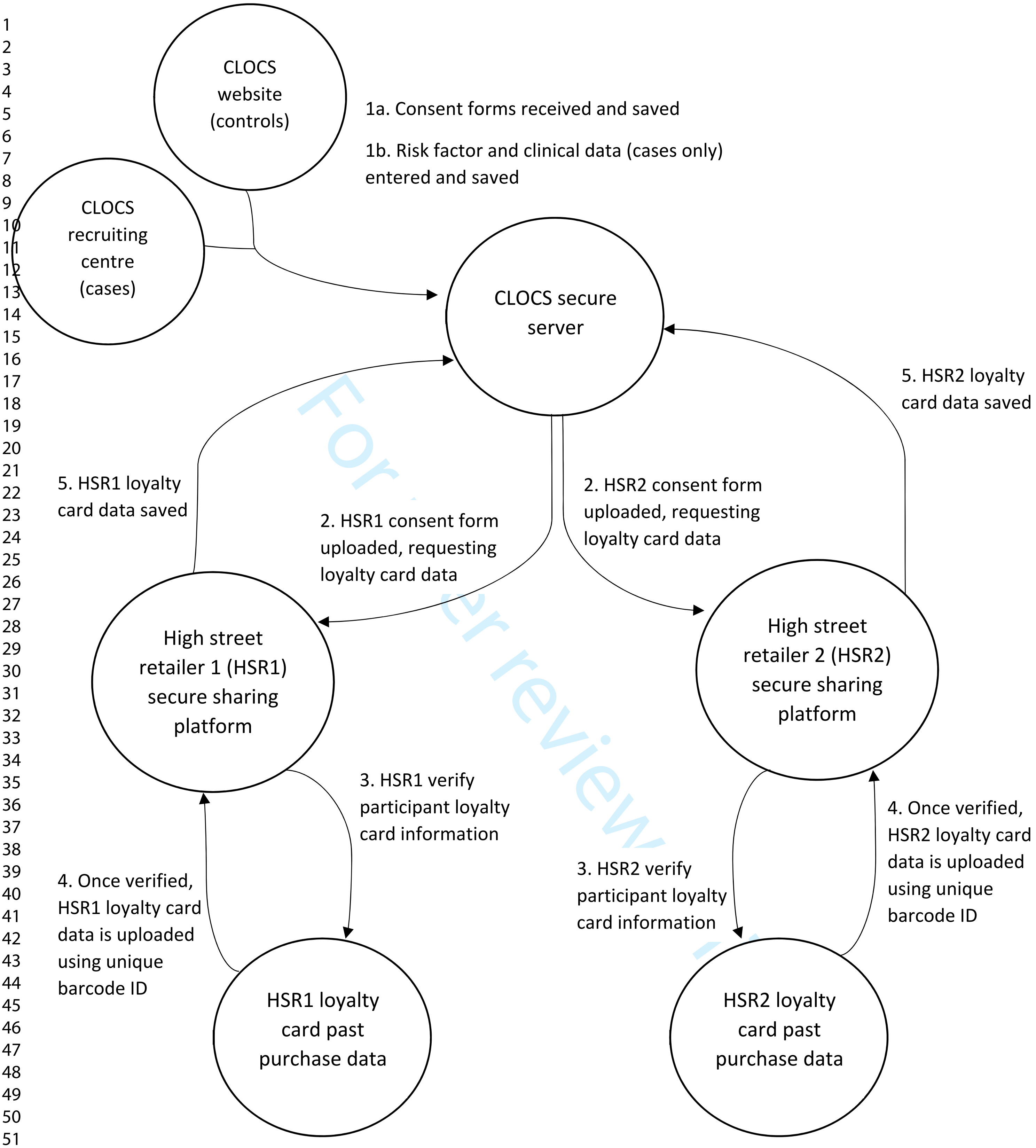

For peer review only

Supplementary Data

Appendix A

Table 1: A list of example target items and behaviours to be analysed using loyalty card data.

**Purchases and behaviours to be explored:**	**Examples:**
Pain relief medications	Paracetamol, ibuprofen, aspirin, heat patch, etc.
Indigestion relief medications	Gaviscon, TUMS, Milk of Magnesia, omeprazole, etc.
Bloating and constipation relief medications	Imodium, Colpermin, Senokot, etc.
Change in diet	Sudden change to 'free from' foods such as gluten free or dairy free, or significant increase in purchasing high fibre foods, etc.

Appendix B

The following STROBE statement for case-control studies is the checklist CLOCS will use to report findings from the case-control study described in this protocol.

STROBE Statement—Checklist of items that should be included in reports of ***case-control studies***

	**Item No**	**Recommendation**
**Title and abstract**	1	(*a*) Indicate the study's design with a commonly used term in the title or the abstract
		(*b*) Provide in the abstract an informative and balanced summary of what was done and what was found
**Introduction**		
Background/rationale	2	Explain the scientific background and rationale for the investigation being reported
Objectives	3	State specific objectives, including any prespecified hypotheses
**Methods**		
Study design	4	Present key elements of study design early in the paper
Setting	5	Describe the setting, locations, and relevant dates, including periods of recruitment, exposure, follow-up, and data collection
Participants	6	(*a*) Give the eligibility criteria, and the sources and methods of case ascertainment and control selection. Give the rationale for the choice of cases and controls
		(*b*) For matched studies, give matching criteria and the number of controls per case
Variables	7	Clearly define all outcomes, exposures, predictors, potential confounders, and effect modifiers. Give diagnostic criteria, if applicable
Data sources/ measurement	8*	For each variable of interest, give sources of data and details of methods of assessment (measurement). Describe comparability of assessment methods if there is more than one group
Bias	9	Describe any efforts to address potential sources of bias
Study size	10	Explain how the study size was arrived at
Quantitative variables	11	Explain how quantitative variables were handled in the analyses. If applicable, describe which groupings were chosen and why
Statistical methods	12	(*a*) Describe all statistical methods, including those used to control for confounding
		(*b*) Describe any methods used to examine subgroups and interactions
		(*c*) Explain how missing data were addressed
		(*d*) If applicable, explain how matching of cases and controls was addressed
		(*e*) Describe any sensitivity analyses

Results

For peer review only - http://bmjopen.bmj.com/site/about/guidelines.xhtml

For peer review only

Participants	13*	(a) Report numbers of individuals at each stage of study—eg numbers potentially eligible, examined for eligibility, confirmed eligible, included in the study, completing follow-up, and analysed
		(b) Give reasons for non-participation at each stage
		(c) Consider use of a flow diagram
Descriptive data	14*	(a) Give characteristics of study participants (eg demographic, clinical, social) and information on exposures and potential confounders
		(b) Indicate number of participants with missing data for each variable of interest
Outcome data	15*	Report numbers in each exposure category, or summary measures of exposure
Main results	16	(*a*) Give unadjusted estimates and, if applicable, confounder-adjusted estimates and their precision (eg, 95% confidence interval). Make clear which confounders were adjusted for and why they were included
		(*b*) Report category boundaries when continuous variables were categorized
		(*c*) If relevant, consider translating estimates of relative risk into absolute risk for a meaningful time period

For peer review only

Other analyses	17	Report other analyses done—eg analyses of subgroups and interactions, and sensitivity analyses
**Discussion**		
Key results	18	Summarise key results with reference to study objectives
Limitations	19	Discuss limitations of the study, taking into account sources of potential bias or imprecision. Discuss both direction and magnitude of any potential bias
Interpretation	20	Give a cautious overall interpretation of results considering objectives, limitations, multiplicity of analyses, results from similar studies, and other relevant evidence
Generalisability	21	Discuss the generalisability (external validity) of the study results
**Other information**		
Funding	22	Give the source of funding and the role of the funders for the present study and, if applicable, for the original study on which the present article is based

*Give information separately for cases and controls.

Note: An Explanation and Elaboration article discusses each checklist item and gives methodological background and published examples of transparent reporting. The STROBE checklist is best used in conjunction with this article (freely available on the Web sites of PLoS Medicine at http://www.plosmedicine.org/, Annals of Internal Medicine at http://www.annals.org/, and Epidemiology at http://www.epidem.com/). Information on the STROBE Initiative is available at http://www.strobe-statement.org.

For peer review only

BMJ Open

Cancer Loyalty Card Study (CLOCS): protocol for an observational case-control study focusing on the patient interval in ovarian cancer diagnosis

Journal:	*BMJ Open*
Manuscript ID	bmjopen-2020-037459.R3
Article Type:	Protocol
Date Submitted by the Author:	06-Jul-2020
Complete List of Authors:	Brewer, Hannah; Imperial College London, Surgery and Cancer<br>Hirst, Yasemin; University College London, Epidemiology and Public Health<br>Sundar, Sudha; University of Birmingham, Cancer Sciences<br>Chadeau-Hyam, Marc; Imperial College London, MRC Centre for Environment and Health; Imperial College London, Epidemiology and Biostatistics, School of Public Health<br>Flanagan, James; Imperial College London, Surgery and Cancer
**Primary Subject Heading**:	Oncology
Secondary Subject Heading:	Epidemiology, Public health
Keywords:	ONCOLOGY, Gynaecological oncology < GYNAECOLOGY, Epidemiology < ONCOLOGY, PUBLIC HEALTH

SCHOLARONE™
Manuscripts

I, the Submitting Author has the right to grant and does grant on behalf of all authors of the Work (as defined in the below author licence), an exclusive licence and/or a non-exclusive licence for contributions from authors who are: i) UK Crown employees; ii) where BMJ has agreed a CC-BY licence shall apply, and/or iii) in accordance with the terms applicable for US Federal Government officers or employees acting as part of their official duties; on a worldwide, perpetual, irrevocable, royalty-free basis to BMJ Publishing Group Ltd ("BMJ") its licensees and where the relevant Journal is co-owned by BMJ to the co-owners of the Journal, to publish the Work in this journal and any other BMJ products and to exploit all rights, as set out in our licence.

The Submitting Author accepts and understands that any supply made under these terms is made by BMJ to the Submitting Author unless you are acting as an employee on behalf of your employer or a postgraduate student of an affiliated institution which is paying any applicable article publishing charge ("APC") for Open Access articles. Where the Submitting Author wishes to make the Work available on an Open Access basis (and intends to pay the relevant APC), the terms of reuse of such Open Access shall be governed by a Creative Commons licence – details of these licences and which Creative Commons licence will apply to this Work are set out in our licence referred to above.

Other than as permitted in any relevant BMJ Author's Self Archiving Policies, I confirm this Work has not been accepted for publication elsewhere, is not being considered for publication elsewhere and does not duplicate material already published. I confirm all authors consent to publication of this Work and authorise the granting of this licence.

For peer review only

TITLE

Cancer Loyalty Card Study (CLOCS): protocol for an observational case-control study focusing on the patient interval in ovarian cancer diagnosis

Short Title

Cancer Loyalty Card Study (CLOCS): protocol for a case-control study

AUTHORS

Hannah R. Brewer[1], Yasemin Hirst[2], Sudha Sundar[3,4], Marc Chadeau-Hyam[5,6] and James M. Flanagan[1]

1. Department of Surgery and Cancer, Imperial College London, London, UK
2. Department of Behavioural Science and Health, University College London, London, UK
3. Pan-Birmingham Gynaecological Cancer Centre, City Hospital, Birmingham, UK.
4. Institute of Cancer and Genomic Sciences, University of Birmingham, Birmingham, UK.
5. MRC Centre for Environment and Health, Imperial College London, London, United Kingdom.
6. Department of Epidemiology and Biostatistics, School of Public Health, Imperial College London, London, United Kingdom.

Address for Correspondence:

Dr. James M. Flanagan

Division of Cancer, Department of Surgery and Cancer,

Faculty of Medicine, Imperial College London

4th Floor IRDB, Hammersmith Campus, Du Cane Road, London W12 0NN

Tel: 020 75941804; email: j.flanagan@imperial.ac.uk

For peer review only

ABSTRACT

Introduction

Ovarian Cancer is the eighth most common cancer in women worldwide, and about one in five women with ovarian cancer do not receive treatment, because they are too unwell by the time they are diagnosed. Symptoms of ovarian cancer are non-specific or can be associated with other common conditions, and women experiencing these symptoms have been shown to self-manage them using over-the-counter medication. Results from a recent proof of concept study suggest there may be an increase in the purchases of pain killers and indigestion medication 10-12 months before ovarian cancer diagnosis. We propose a case-control study, as part of a larger project called the Cancer Loyalty Card Study (CLOCS), to investigate whether a significant change in medication purchases could be an indication for early signs of ovarian cancer, using data already collected through store loyalty cards.

Methods and Analysis

Using a retrospective case-control design, we aim to recruit 500 women diagnosed with ovarian cancer (cases) and 500 women without ovarian cancer (controls) in the United Kingdom who hold a loyalty card with at least one participating high street retailer. We will use pre-existing loyalty card data to compare past purchase patterns of cases with those of controls. In order to assess ovarian cancer risk in participants and their purchase patterns, we will collect information from participants on ovarian cancer risk factors and clinical data including symptoms experienced before diagnosis from recruited women with ovarian cancer.

Ethics and dissemination

CLOCS was reviewed and approved by the North West - Greater Manchester South Research Ethics Committee (19/NW/0427). Study outcomes will be disseminated through academic publications, the study website, social media, and a report to the research sites that support the study once results are published.

Study Registration Numbers

CLOCS is registered with ISRCTN (14897082), NIHR portfolio (CPMS 43323), and clinicaltrials.gov (NCT03994653).

Keywords:

Ovarian cancer, early detection, purchase behaviour, observational case-control study, commercial data

For peer review only - http://bmjopen.bmj.com/site/about/guidelines.xhtml

Strengths and limitations of this study

- This study is a novel approach to investigate whether routinely collected commercial purchase data can be used to reduce delays in ovarian cancer diagnosis.
- Ovarian cancer advocates have been involved in the conception, design and conduct of the study.
- A key strength of the study is the detailed information available for purchases recorded for up to 7 years prior to enrolment.
- A key limitation is that loyalty card holders may not use their cards for every purchase, may shop at other stores not being accessed in this study and may be purchasing items for other family members.
- In general, due to the retrospective nature of case-control studies, there is potential for the misreporting of exposures in the risk factor questionnaire by cases and controls (recall bias).

INTRODUCTION

Approximately 7,200 new cases of ovarian cancer are diagnosed each year in the UK, and with over 4,200 women dying from the disease each year, it is a particularly lethal form of cancer[1]. Diagnosing ovarian cancer in primary and secondary care settings among symptomatic patients is a challenge due to their low positive predictive value of symptoms and symptom presentation at various stages of the ovarian cancer prognosis[2, 3]. Symptoms, such as increased abdominal size, pelvic pain, abdominal pain and bloating, feeling full quickly and difficulty eating have been identified as high risk symptoms but equally it has been reported that the absence of a symptom does not help ruling out ovarian cancer[4, 5]. For patients whose symptoms do not warrant an immediate referral earlier presentation in primary care is crucial to reduce delays in diagnosis and subsequent treatment.

However, symptom recognition and help-seeking in primary care are also not without challenges. While in some cases patients may present very quickly, for some patients it could take up to 12 months before they seek any medical help[6]. It has been shown that greater awareness of symptoms alone does not facilitate help seeking for ovarian cancer because symptoms are often perceived as vague or trivial[7, 8]. The major reasons for not presenting to the primary care are often highlighted as “not wanting to waste the GP’s time”[9],

For peer review only - http://bmjopen.bmj.com/site/about/guidelines.xhtml

normalisation of these symptoms[10], and few women believing that their symptom(s) might be a sign of cancer[11]. Women might choose to self-manage such symptoms by seeking advice from sources other than a primary care physician, e.g. pharmacists, family, internet, and using over-the-counter medication[12] which may also have been moderated by other factors such as access to healthcare, sociodemographic factors and other competing priorities, e.g. work and care responsibilities[7-11].

There are currently no organised screening programmes offered to women that could help detect ovarian cancer at an earlier stage, and there is little evidence for effective interventions aiming to improve symptom recognition and help-seeking.

Therefore, novel methods are needed to improve earlier detection of low-volume disease or earlier stage to improve survival[13]. A recent proof of concept study investigated whether purchase information collected through the high street retailers' loyalty cards (e.g. store points cards) could be used to identify women's self-care behaviours prior to presenting to health care professionals. This study included data from a major high street retailer's loyalty card of women who have previously been diagnosed with ovarian cancer and consented their past purchase data to be provided to the researchers. The study suggested that there may be an increase in the purchases of pain killers and indigestion tablets up to 10-12 months before diagnosis[14]. This proof-of-concept study provided a new model of investigation for symptom recognition and help-seeking among ovarian cancer patients. It highlighted potential strengths and limitations in its methodology for future research. Building on these results, self-care behaviours of ovarian cancer patients will be further investigated with a case-control study within a larger project called Cancer Loyalty Card Study (CLOCS). CLOCS hypothesises that a significant change in purchasing behaviour, primarily an increase in self-medication or self-care behaviours to treat symptoms prior to presentation in primary care (see Figure 1), could be an indication for early signs of ovarian cancer.

Study aims

The primary aim of this case-control study is to investigate pre-diagnosis purchasing behaviours of ovarian cancer patients (cases) with women without a previous ovarian cancer diagnosis (controls) using data collected through two major high street retailers' loyalty card schemes. We will investigate purchase behaviour of items that are suspected to treat symptoms of ovarian cancer (e.g. painkillers, indigestion tablets) as well as a change in frequency or quantity of other purchased items. The secondary aim of CLOCS will be to

For peer review only - http://bmjopen.bmj.com/site/about/guidelines.xhtml

investigate whether or not a purchase threshold as an "alert" about potential ovarian cancer symptoms in individuals can be possible using loyalty card data and determining the predictive utility of purchasing behaviours in the early detection of ovarian cancer based on ovarian cancer risk. This would be important to inform if future interventions would be possible using loyalty card data. The findings from this retrospective study will need to be validated in a prospective cohort study prior to any clinical impact being realised. However, the clinical impact of this study for ovarian cancer diagnosis may involve a number of potential interventions. Future interventions could include an alert sent to individuals who exceed the "alert threshold" to visit a GP to discuss their symptoms, or a potential public health message about those purchase thresholds and self-treatment of ovarian cancer symptoms.

METHODS AND ANALYSIS

Study design and setting

CLOCS will use a retrospective case-control design to compare past purchase patterns of women with and without ovarian cancer using already existing data gathered by two major high street retailers, one of which is a health and beauty retailer and the other a groceries and general merchandise retailer. Furthermore, we will collect data on risk factors for ovarian cancer, symptoms experienced before diagnosis and clinical data from ovarian cancer patients using self-reported questionnaires.

CLOCS will be recruiting across the United Kingdom from at least 40 National Health Service (NHS) Trusts and Hospitals and recruit healthy volunteers using online recruitment.

Study timeline

The CLOCS case-control study will take place from 1st November 2019 until 1st February 2022.

Participants

Inclusion/Exclusion Criteria

Cases

Women, at least 18 years old, diagnosed and living with any type of ovarian cancer (including all subtypes of epithelial ovarian cancer high-grade serous, low-grade serous, endometrioid, clear cell, mucinous, borderline, and other subtypes) within the previous 2

For peer review only - http://bmjopen.bmj.com/site/about/guidelines.xhtml

years from the recruitment date who own at least one participating high street retailer loyalty card are eligible to join CLOCS case-control study as cases.

There are no further clinical criteria for patients to be excluded from this study. Patients' usual care will continue to be provided by the health care professionals, and CLOCS participation does not exclude patients from participating in other clinical or non-clinical studies.

Controls

Women, at least 18 years old, who have not been diagnosed with ovarian cancer and own at least one of the loyalty cards from the participating retailers are eligible to join the CLOCS as controls. We are not excluding women with previous cancer diagnosis, or women who may have had their ovaries removed for reasons other than cancer; however, these will be considered when cases are matched with controls.

Recruitment

The CLOCS case-control study recruitment process is outlined in figure 2 and described further detail below.

Cases

Eligible ovarian cancer patients will be recruited by members of their healthcare teams at approximately 40 NHS Trusts and Hospitals across the United Kingdom that have agreed to approach patients at clinics. These healthcare teams are registered as research sites and will have a principal investigator (PI) leading the recruitment to CLOCS. The PI and the research nurses will facilitate recruitment by identifying patients when they think it is suitable to approach patients based on patients' overall well-being at a suitable time (e.g. at waiting areas, during chemotherapy sessions), then will provide patients with the study information to consider participation. Patients will be given a paper-based participant pack that includes study consent form including consents for providing access to data collected by the retailers, self-report questionnaire, and a clinical questionnaire to be completed by the clinical staff facilitating the recruitment. If they wish to participate, patients can complete all the study documents at the recruitment site and post it back to the research team using the free-post envelope provided in the study pack, or they are also given the option to consider participation at home provided that the clinical questionnaire has been completed by a member their clinical team. The research sites will not need to record any data from the participants. The data collection from the cases will be a one-off process and no further contact with the patient is required for the study.

For peer review only - http://bmjopen.bmj.com/site/about/guidelines.xhtml

Controls

The control participants to the study will be recruited through the study website (www.clocsproject.org.uk) where data collection will be safeguarded using an encrypted web form that includes study consent and the self-report questionnaire. The website has been developed and maintained by NDP limited. The questionnaire for women without ovarian cancer will be confined to risk factors. The online participation will take up to 15 minutes and data will be transferred immediately to the secure CLOCS server (see figure 3). The website approach was adopted as one of the outcomes of the proof-of-concept study. It aims to provide a transparent environment where potential participants can make informed decisions about participation in CLOCS, and the data risks are minimised using encryption. We have carefully designed the website to inform the general public about how we will process their data in CLOCS, how they can withdraw from the study if they wish to do so and use it as a platform to provide feedback as we progress in the larger CLOCS project. The research team will use several methods to recruit control participants to the study which will include a press release carried out by the project funders and the Imperial College London public engagement team, dissemination of study flyers and leaflets to patients in participating NHS recruitment clinics, and social media marketing techniques on Twitter and Facebook using paid advertisement and unpaid methods. The application of the social media recruitment methods will allow researchers to reach a larger cohort in the UK. The application of both paper-based and online recruitment methods will be carried out throughout CLOCS and will be recorded. The efficacy of these methods will be reported in peer-reviewed journals to inform online research recruitment to future case-control studies.

Data sources and variables included

Loyalty Card Data (For cases and controls)

CLOCS participants will hold a loyalty card at one or both participating retailers, including two health, beauty and/or grocery retailers that can be accessed by the UK population within all precincts allowing the study to assess purchases of painkillers and indigestion medication as well as eating habits. The study will not include prescription-based pharmacy data. Once participants consent for the CLOCS team to request their loyalty card data from the retailers on their behalf, the study will include individuals' past purchase data from up to six years prior to the date of study consent. The purchase data will include each individual purchase, date of purchase, the location (i.e. store postcode), and the product categorisation that is provided by the retailers. There will be no additional individual information recorded by the

For peer review only

For peer review only - http://bmjopen.bmj.com/site/about/guidelines.xhtml

study team from this dataset and no additional individual information will be requested from the retailers. Please note that at this stage of the study, we will not be naming the high street retailers in publications and in our direct communications, but they are named to the potential participants when they agree to take part.

Ovarian Cancer Risk Questionnaire (For Cases and Controls)

In order to stratify the purchase differences between cases and controls based on ovarian cancer risk, we designed a questionnaire on ovarian cancer risk factors that will allow us to determine individuals' ovarian cancer risk. Both cases and control participants will be asked to complete a short questionnaire about ethnicity, marital status, and the following well-established ovarian cancer risk factors: body mass index, age at menarche, menopausal status, age at menopause, parity, breastfeeding, hysterectomy, tubal ligation, cancer history, endometriosis, aspirin use, oral contraceptive use, hormone replacement therapy (HRT), family history of ovarian and breast cancers, vaping, and cigarette smoking[15]. These factors will be used to assess ovarian cancer risk among participants to distinguish high-risk and low-risk individuals and subsequently observe patterns in purchase history stratified by risk levels.

The questionnaire also includes questions about the symptoms experienced (if any) and number of visits to the general practitioner in the year leading up to cancer referral or diagnosis which were adopted from the proof-of-concept study[14].

The final section of the questionnaire contains questions about which food and pharmacy stores participants shop at regularly, at which stores they own a loyalty card, and frequency of use of those loyalty cards.

Clinical Questionnaire (Cases only)

Women with an ovarian cancer diagnosis will also have a clinical questionnaire completed by their healthcare team. This will provide the objective timeline and pathway for diagnosis (e.g. GP referral, emergency presentation, other), details on the type (high-grade serous, low-grade serous, endometrioid, clear cell, mucinous, borderline, and other), stage (I, II, III, or IV), grade of ovarian cancer at diagnosis (grade 1 or well differentiated, 2 or moderately differentiated, 3 or poorly differentiated/undifferentiated), surgical outcome if any (optimal debulking, sub-optimal debulking, or no surgery), and BRCA status if the participant has been tested (BRCA1 positive, BRCA2 positive, both negative, awaiting result). We will use this information in risk assessment and to stratify the loyalty card data of cases and controls based on the time of diagnosis, and observe purchase history stratified by type, stage, and grade of ovarian cancer diagnoses.

For peer review only - http://bmjopen.bmj.com/site/about/guidelines.xhtml

Data collection processes (see figure 3)

In the next section we will describe, how we will be collecting and processing data in the CLOCS case-control study.

1) Data Acquisition

Upon choosing to participate, both cases and controls will need to complete a consent form to participate in CLOCS and return it to the CLOCS research team either by post (cases only) or the CLOCS website (controls only). The CLOCS consent form includes two sections. The first section details consent to take part in CLOCS, and the second section is for participants to consent to the CLOCS research team to request their data from high street retailers on their behalf. The consents for loyalty cards include the exact name of the individual and the exact membership number as written on their card. All individual consents including scanned copies of paper consents will be saved in the secure and encrypted server based in the "Secure Enclave" at Imperial College London. Participant loyalty card details will be entered in to a comma-separated values (CSV) file and encrypted for secure data request from retailers using a secure file transfer protocol (SFTP).

2) Data Extraction from the high street retailers

The CLOCS team will send the CSV file that contains a unique study ID, name of the individual and the exact loyalty card number specific to the retailer using the secure file transfer. The respective high street retailer will validate the participant information from the consent form with their loyalty card data. If loyalty card details cannot be validated, the consents and any information from those individuals who have not consented to be re-contacted will be excluded from the study and deleted immediately. High street retailers will then generate a dataset for all consenting individuals and will share the past purchase data with the CLOCS team using the SFTP. Depending on the high street retailer's records and when the participant obtained the loyalty card, up to 7 years of participants' past purchasing data prior to the date of recruitment will be shared.

3) Data Merge

The CLOCS team will receive the encrypted participant purchase history from the high street retailers through the SFTP, save, and store all data to a secure and encrypted server

For peer review only - http://bmjopen.bmj.com/site/about/guidelines.xhtml

based in the Secure Enclave at Imperial College London, described in *Data management*. All data will be pseudonymised and analysed on the secure server.

4) Data Deletion

The information relating to the participants' consents for providing past purchase data will be retained in line with each high street retailers' data retention policy. Ten years after the study is complete, all paper-based documents will be shredded and destroyed in accordance with Imperial College London's data deletion and retention processes. Electronically held identifiable data including individual consent and contact details will be kept for 10 years after the study is complete and will be deleted from the secure server in accordance with Imperial College London's data deletion and retention processes. Past purchase data will be kept until the results are published or five years from the date of participation in the study, whichever comes sooner, before it will be anonymised and the link to all identifiable data will be deleted. During the lifetime of the study, participants are given the option to withdraw consent which will mean that all data associated with that individual will be deleted and records will be removed from CLOCS.

Sample size and statistical power

Based on the modelled data from the pilot study[14], we hypothesise that we could detect a difference in purchase proportions of 8.3% at 3 months prior to diagnosis, 6.3% at 6 months, 4.3% at 9 months and 2.3% at 12 months prior to diagnosis between cases and controls. Using the two-sample test for proportions the minimum group size to detect these differences at 80% power and α=0.05 would be 125 (-3 months), 185 (-6 months), 328 (-9 months) and 969 (-12 months). We modelled the power versus time prior to diagnosis and chose 10 months as our target to retain sufficient power for larger effect sizes at earlier time points. Therefore, in the final analysis we will have >80% power at α=0.05 to detect an increase in proportion of purchases in cases (>4.7%) vs controls (1.4%) at 10 months prior to diagnosis, based on the modelled data, with a minimum n=431 cases. We aim to recruit 500 participants to account for 10-15% of very low frequency card users. We will compare proportions using Fishers Exact Test comparing target (items suspected to be used to treat ovarian cancer symptoms) and non-target (all other items) purchases in cases at each time point prior to diagnosis compared with the average monthly proportions in the controls. Target items for analysis will include but will not be limited to medications for pain, indigestion, and bloating

For peer review only - http://bmjopen.bmj.com/site/about/guidelines.xhtml

relief (see table 1 in Appendix A of the supplementary material for a list of example purchases). We will report proportions, 95% confidence intervals and *P*-values.

Statistical Methods

We will use descriptive statistics to provide details on the characteristics of the cases and controls by sociodemographic (e.g. age, ethnicity) and by risk factors (e.g. parity, oral contraceptive use, etc.). We will report feasibility factors such as number of people who had both, or one loyalty card, and number of years of data included in the study.

Cases and controls will be matched 1:1 by age (5-year age groups) and ethnicity. Ethnicity will be matched based on the guidance from the Office for National Statistics[16]. We will aim to define the time by which cases and controls become statistically significantly different in their purchase behaviours using a multivariable, conditional logistic regression model with ovarian cancer as the outcome and "purchase proportion" at different time points to diagnosis as the exposure, adjusting for stage and histology and other potentially confounding variables collected from the risk factor questionnaire (including body mass index, age at menarche, menopausal status, age at menopause, parity, breastfeeding, hysterectomy, tubal ligation, cancer history, endometriosis, aspirin use, oral contraceptive use, hormone replacement therapy (HRT), family history of ovarian and breast cancers, vaping, and cigarette smoking[15]). We will compare the proportions of purchases in cases to controls, stratified by risk quintiles defined by the risk model (estimated n=86 per quintile) and by histology (with expected proportions of high-grade serous, mucinous cases, endometrioid, clear cell, and low-grade serous cases). Analyses will be performed for different types of medications separately and combined (e.g. a comparison of case-control purchase proportion of painkillers alone and indigestion medications alone, and then of painkillers and indigestion medications combined).

Established risk factors have been reported to modestly discriminate between cases and controls (AUC=0.66)[17], and polygenic risk scores only slightly improves these performances. This model has however been proposed to improve epithelial ovarian cancer detection with risk stratified screening[18]. We will establish the risk profile, a mathematical summation of all of the risk factors, of each participant based on the published method [17]. Our study will be underpowered to establish a difference between high and low risk groups (20% of subjects per quintile of risk), it is therefore exploratory. We will estimate the predictive values of this "early diagnosis" test for each stratum. If this analysis indicates a

For peer review only - http://bmjopen.bmj.com/site/about/guidelines.xhtml

potential difference, then that will guide future studies that will be sufficiently powered to address this.

We will conduct exploratory analyses investigating other methods leveraging the auto correlation in the longitudinal data. Specifically, we will explore the evolutions of individual frequency of purchases and purchase trajectories, to identify purchase patterns (e.g. increasing purchases before diagnosis, purchase peaks, etc.), that are indicative/predictive of the disease onset and of the time-to-onset.
All analyses will be done using the statistical software R.

Interim Analysis

An interim analysis will be conducted after six months of CLOCS recruitment to provide an initial exploratory analysis of the data and assess the demographics of CLOCS participants. This will help determine whether recruitment will need to target specific demographics (e.g. age groups) in order to ensure there is a representative distribution of ages and ethnicities for the matched analysis.

Study risks and sources of bias

There are no clear risks for participants in the CLOCS. However, it may be emotional or distressing for patients to think about cancer and the first time they noticed some changes in their body (i.e. symptoms). The CLOCS team intended to design the survey questions in the most sensible and sensitive way to ensure that there are no negative effects of this study on patients' well-being. All participants are advised to see a medical professional in the information sheet if they are worried about a symptom or sign that they might have been experiencing.
For many loyalty card holders, purchases will be for themselves and other individuals in their household. The CLOCS team will not be able to quantify what purchases are consumed by the participant and by other members of the household, but the team will be able to make assumptions to adjust accordingly.
The CLOCS team acknowledges that not all participants will have used their loyalty card with every purchase for various reasons (e.g. card not with them at time of purchase, in a hurry, etc.) and that participants will have made purchases at other shops not recorded with their loyalty cards observed in this study. For this reason, the risk factor questionnaire asks participants to indicate how often they use their loyalty card when shopping, at which stores

For peer review only

they shop most regularly and own a loyalty card, so that these can be taken account at the analysis stage.

It is possible that CLOCS participants could have other comorbidities, and their over-the-counter purchases might be used to treat symptoms from those instead of symptoms of ovarian cancer. If women have been diagnosed with comorbidities (e.g. asthma, diabetes, osteoporosis, depression, etc. [19]), we would assume that they might already be receiving prescribed medication to treat these from their healthcare providers. If they are using prescribed medication, then loyalty card data does not include information about prescriptions. Common comorbidities that are not yet diagnosed or that do not require prescription medication are expected to be present in similar frequencies among CLOCS participants with and without ovarian cancer. The risk factor questionnaire will address some of these comorbidities, which will allow us to adjust for them in the analysis.

It is also possible that ovarian cancer patients may have already been seen by a health care professional for their symptoms and may have been prescribed medication for reasons other than ovarian cancer, e.g. urinary tract infection, irritable bowel syndrome, prior to being referred for suspected ovarian cancer. However, access to prescribed medication information would require access to primary care records and not included in this study. It would also mean that patients have already sought help for their symptoms and the patient interval phase for symptom appraisal before help-seeking would have been finalised. We will control for this by adjusting for the self-reported number of visits to the GP leading up to the diagnosis. This would also be discussed as a potential limitation, and we will be cautious in our evaluations of the results if we observe a reduction in risk related purchases close to the date of diagnosis.

CLOCS limits participation to women who own a loyalty card from at least one of the participating retailers. Approximately 73% of women aged 50-80 years in the UK have a loyalty card to one of the participating high street retailers, and therefore the cardholders in CLOCS should be representative. Nevertheless, there will be selection bias against those who do not hold a loyalty card.

In any retrospective case-control study, there is the potential for recall bias. Participants with ovarian cancer could report experiencing more symptoms more frequently than may have occurred, but for those who report they self-managed these symptoms, the CLOCS team will be able to observe loyalty card purchase data to validate the amount and frequency of self-medication prior to diagnosis.

Data management

For peer review only - http://bmjopen.bmj.com/site/about/guidelines.xhtml

The CLOCS research team will act as *data processors* when requesting data from high street retailers and the high street retailers would be *data controllers*. CLOCS will become the data controller for the self-reported questionnaire data and the clinical questionnaire data. The consenting participants will be *data owners* and will have the right to withdraw their consent from the study at any time without giving any reason to the study team.

Only the CLOCS research team will have access to the paper-based consent forms which will be kept in a locked cupboard and server in a locked room and secure building that requires security badge access to enter. The data will be analysed in a secure environment with access limited to the research team. Participants will be given a unique study ID barcode which means all datasets will be pseudonymised.

All data will be stored and processed in Imperial College's ISO27001 certified secure environment – the "Secure Enclave". This is a fully managed infrastructure and secure environment providing high availability, resilience and business continuity through multiple servers, back-ups and disaster recovery measures. A robust data security model has been designed to protect sensitive personal and medical data from the potential risk of unauthorised access or distribution. No one other than the research team will have access to the research dataset.

The CLOCS website is compliant with the high level of data security required by Imperial College London and General Data Protection Regulation (GDPR). The data collected through the website only resides in the Secure Enclave and not in the browser or website. It also uses Public Key Encryption, which encrypts data from the site that can only be unlocked once within the enclave. These requirements are equalled by support for the user to complete the online survey, by using content and interfaces that are easy to use, especially on mobile phones.

We have strict data processing agreements in place with our commercial partners to ensure that data is protected at all times. Commercial partners will not have access to the study data and will not financially benefit from this project.

Patient and public involvement

Ovarian cancer patients and members of the public have been involved in the development of the CLOCS from the beginning, participating in the proof-of-concept study that assessed the general public's acceptability of the use of commercial and health data linkages for cancer symptom surveillance[14]. Two ovarian cancer patient advocates who participated in the pilot study have taken part in research meetings developing this case-control study and reviewed

For peer review only - http://bmjopen.bmj.com/site/about/guidelines.xhtml

study documentation. They have been and will continue to be involved in ongoing development of CLOCS methods and dissemination of the research results through social media, media outlets and patient groups. The questionnaire for women without ovarian cancer has also been reviewed and approved by members of the public.

We also use our website for providing information to the patients and public. For instance, all research goals, how we will be analysing data, confidentiality and data security are all detailed for the lay population. There is an animation hosted on our website that is aiming to explain the study in simpler words. All materials are reviewed and approved by patient advocates. We carry out annual research meetings with stakeholders including research nurses, patients, academic collaborators, retailers, funders and public to provide updates and receive feedback on this project.

ETHICS AND DISSEMINATION

CLOCS was reviewed and approved by the North West - Greater Manchester South Research Ethics Committee (19/NW/0427). The study is registered as an NHIR portfolio study (Ref: CPMS 43323), with the ISRCTN (14897082), and is registered on clinicaltrials.gov (NCT03994653). This paper describes the ethics approved protocol dated 8 August 2019 (version 2).

We will disseminate the outcomes of the study using academic publications, the study website (clocsproject.org.uk), social media (@CLOCS_Imperial), and send a report to the research sites that supported the study once the results are published. We will aim to publish the results in a peer-reviewed journal in accordance with the STROBE statement for case-control studies[20]. The STROBE statement we intend to use when reporting the results from this case-control study is listed in Appendix B in the supplementary material. We will also use the study website to inform the public about the study outcomes.

Data Sharing Policy

All access to the CLOCS data collected from the retailers will be restricted and will not be shared with other researchers due to the data sharing agreements with the retailers. However, we do encourage other researchers to contact us for collaborations. If they are interested, they are given the opportunity to review the metadata by directly contacting us and submit a research proposal where the data analyses will be carried out by a member of the CLOCS team. All proposals will be reviewed case by case by the PI and the rest of the research team.

For peer review only - http://bmjopen.bmj.com/site/about/guidelines.xhtml

Authors' contributions:

JMF and YH conceived the study. HRB wrote the first draft of the manuscript. YH, JMF, SS and MC-H commented and reviewed the final draft of the manuscript. All authors agree on the final version of the protocol.

Funding statement:

Cancer Loyalty Card Study (CLOCS) is funded by Cancer Research UK (CRUK) Early Detection Project Grant (C38463/A26726). YH and HRB are funded by CRUK.

Competing interests statement

The authors declare no competing interests.

Acknowledgements

We thank NDP Studio Limited for their support and development of the study website (www.clocsproject.org.uk). We thank our patient and public representatives for reviewing the study materials and providing guidance on study acceptability from the conception of CLOCS to data collection. The authors acknowledge infrastructure and recruitment support from the Imperial Experimental Cancer Medicine Centre, Cancer Research UK Imperial Centre, the National Institute for Health Research Imperial Biomedical Research Centre, and the Ovarian Cancer Action Research Centre. We thank Boots UK employees who facilitated the project for their support and guidance during the development of this project. We thank Eric Johnson for guidance on data security and GDPR.

Figure Legends

Figure 1: Pathway to diagnosis highlighting the patient interval that CLOCS will investigate.

Figure 2: CLOCS recruitment process.

Figure 3: CLOCS data flowchart.

For peer review only

REFERENCES

1. Cancer Research UK. Ovarian cancer statistics. https://www.cancerresearchuk.org/health-professional/cancer-statistics/statistics-by-cancer-type/ovarian-cancer. Accessed 9 Apr 2019.

2. Funston G, Van Melle M, Baun M, Jensen H, Helsper C, Emery J, et al. Variation in the initial assessment and investigation for ovarian cancer in symptomatic women: A systematic review of international guidelines. BMC Cancer. 2019.

3. National Institute for Health and Care Excellence. Ovarian cancer: recognition and initial management. Clinical guideline [CG122]. 2011.

4. Ebell M, Culp M, Radke T. A Systematic Review of Symptoms for the Diagnosis of Ovarian Cancer. American Journal of Preventive Medicine. 2016;50:384–94. doi:10.1016/j.amepre.2015.09.023.

5. Goff B. Symptoms associated with ovarian cancer. Clin Obstet Gynecol. 2012;55:36–42.

6. Hamilton W, Peters TJ, Bankhead C, Sharp D. Risk of ovarian cancer in women with symptoms in primary care: Population based case-control study. BMJ. 2009;339:616.

7. Brain K, Smits S, Simon A, Forbes L, Roberts C, Robbé I, et al. Ovarian cancer symptom awareness and anticipated delayed presentation in a population sample. BMC Cancer. 2014.

8. Low E, Waller J, Menon U, Jones A, Reid F, Simon A. Ovarian cancer symptom awareness and anticipated time to help-seeking for symptoms among UK women. J Fam Plan Reprod Heal Care. 2013.

9. ICBP. The international cancer benchmarking partnership evidence for policy and practice October 2016. 2016.

10. Low E, Whitaker K, Simon A, Sekhon M, Waller J. Women's interpretation of and responses to potential gynaecological cancer symptoms: A qualitative interview study. BMJ Open. 2015;5.

11. Whitaker K, Winstanley K, Macleod U, Scott S, Wardle J. Low cancer suspicion following experience of a cancer "warning sign." Eur J Cancer. 2015;51:2473–9.

12. Jayde V, Boughton M. The diagnostic journey of ovarian cancer: A review of the literature and suggestions for practice. Contemporary Nurse. 2012.

13. Gilbert L, Basso O, Sampalis J, Karp I, Martins C, Feng J, et al. Assessment of

For peer review only

For peer review only - http://bmjopen.bmj.com/site/about/guidelines.xhtml

symptomatic women for early diagnosis of ovarian cancer: results from the prospective DOvE pilot project. Lancet Oncol. 2012;13:285–91. doi:10.1016/S1470-2045(11)70333-3.

14. Flanagan J, Skrobanski H, Shi X, Hirst Y. Self-Care Behaviors of Ovarian Cancer Patients Before Their Diagnosis: Proof-of-Concept Study. JMIR Cancer. 2019;5:e10447. doi:10.2196/CANCER.10447.

15. Reid B, Permuth J, Sellers T. Epidemiology of ovarian cancer: a review. Cancer Biol Med. 2017;14:9–32. doi:10.20892/j.issn.2095-3941.2016.0084.

16. Office for National Statistics. Ethnic group, national identity and religion, Measuring equality: A guide for the collection and classification of ethnic group, national identity and religion data in the UK. 2016. https://www.ons.gov.uk/methodology/classificationsandstandards/measuringequality/ethnicgroupnationalidentityandreligion#toc.

17. Clyde M, Palmieri Weber R, Iversen E, Poole M, Doherty J, Goodman M, et al. Risk Prediction for Epithelial Ovarian Cancer in 11 United States-Based Case-Control Studies: Incorporation of Epidemiologic Risk Factors and 17 Confirmed Genetic Loci. Am J Epidemiol. 2016;184:555–69.

18. Meisel SF, Side L, Fraser L, Gessler S, Wardle J, Lanceley A. Population-based, risk-stratified genetic testing for ovarian cancer risk: A focus group study. Public Health Genomics. 2013;16:184–91.

19. Minlikeeva AN, Freudenheim JL, Eng KH, Cannioto RA, Friel G, Szender JB, et al. History of comorbidities and survival of ovarian cancer patients, results from the ovarian cancer association consortium. Cancer Epidemiol Biomarkers Prev. 2017;26:1470–3.

20. von Elm E, Altman DG, Egger M, Pocock SJ, Gøtzsche PC, Vandenbroucke JP. The Strengthening the Reporting of Observational Studies in Epidemiology (STROBE) statement: guidelines for reporting observational studies. Lancet. 2007.

For peer review only - http://bmjopen.bmj.com/site/about/guidelines.xhtml

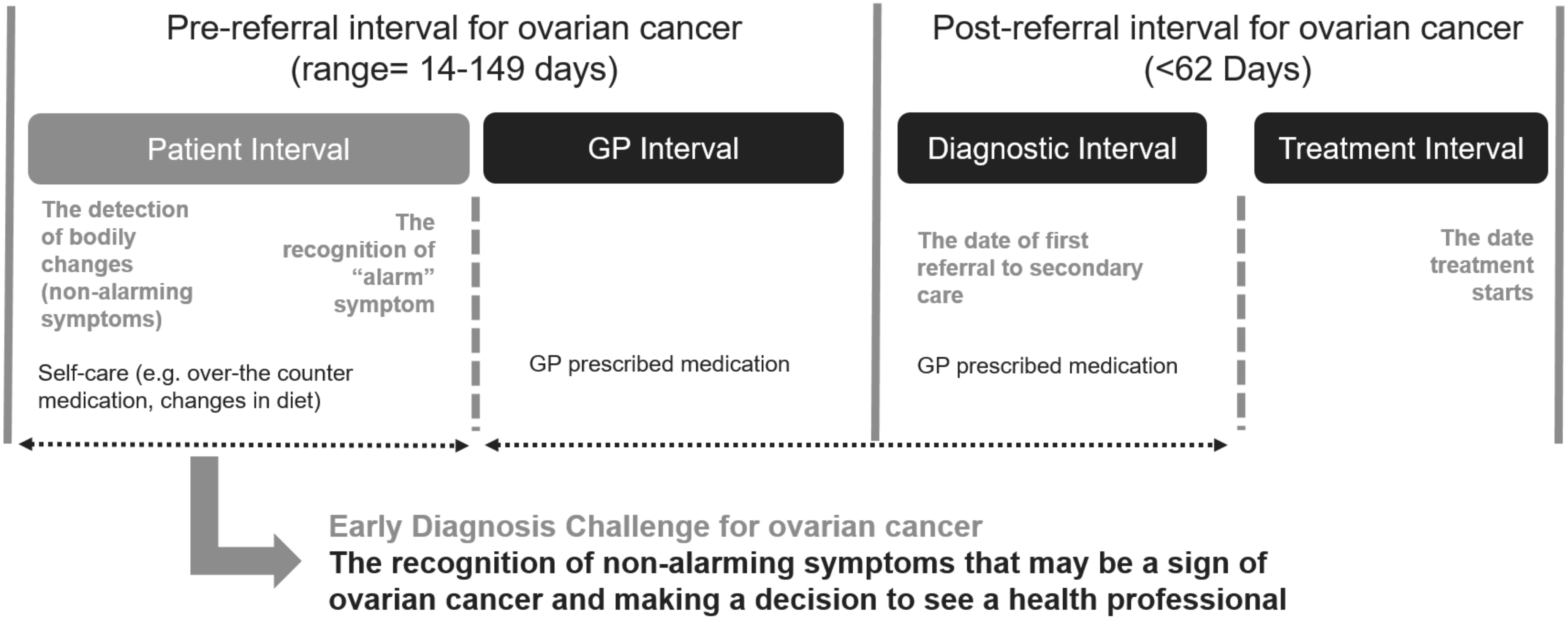
Pre-referral interval for ovarian cancer
(range= 14-149 days)
Post-referral interval for ovarian cancer
(<62 Days)
Patient Interval
GP Interval
Diagnostic Interval
Treatment Interval
The detection of bodily changes (non-alarming symptoms)
The recognition of "alarm" symptom
The date of first referral to secondary care
The date treatment starts
Self-care (e.g. over-the counter medication, changes in diet)
GP prescribed medication
GP prescribed medication
Early Diagnosis Challenge for ovarian cancer
The recognition of non-alarming symptoms that may be a sign of ovarian cancer and making a decision to see a health professional

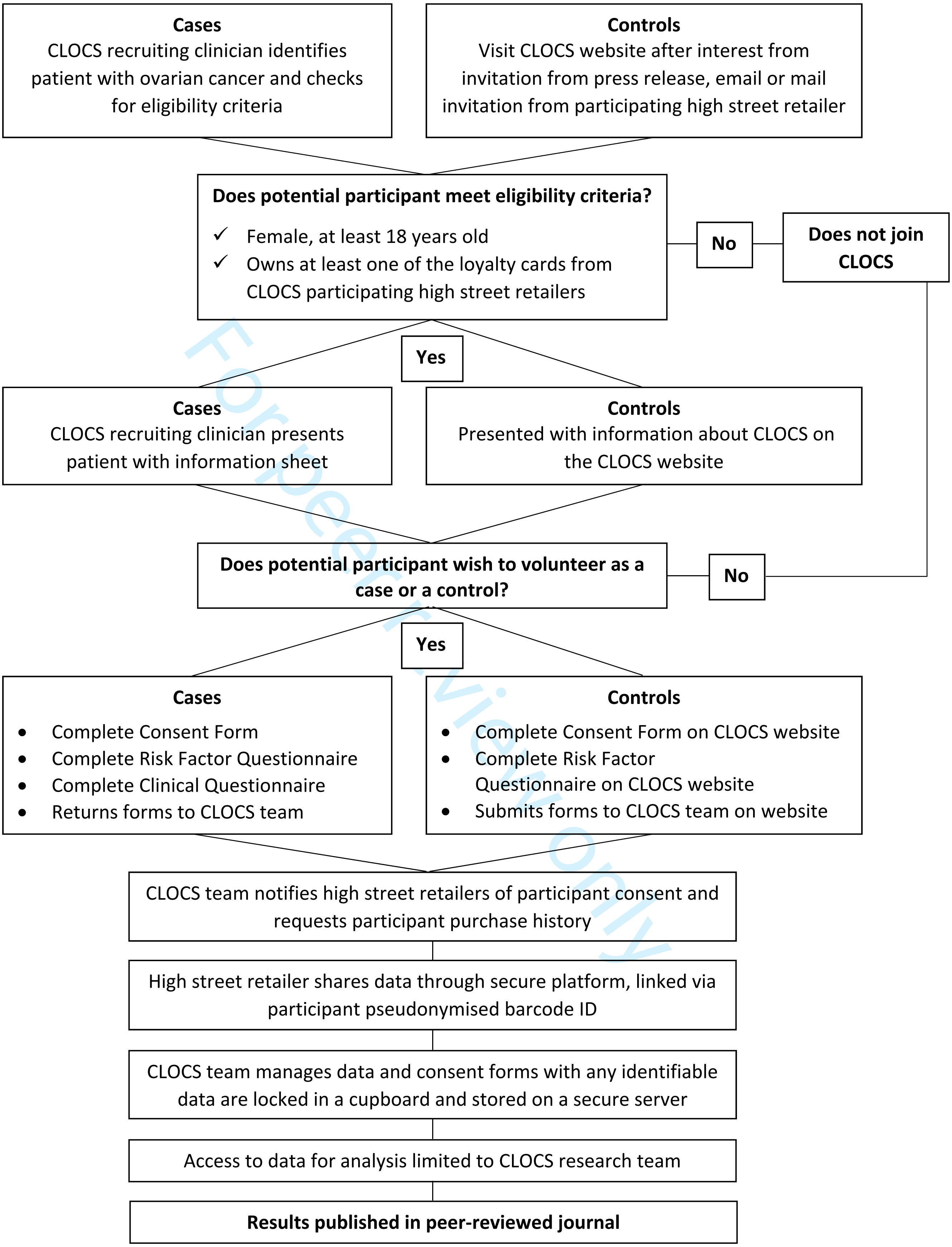

For peer review only

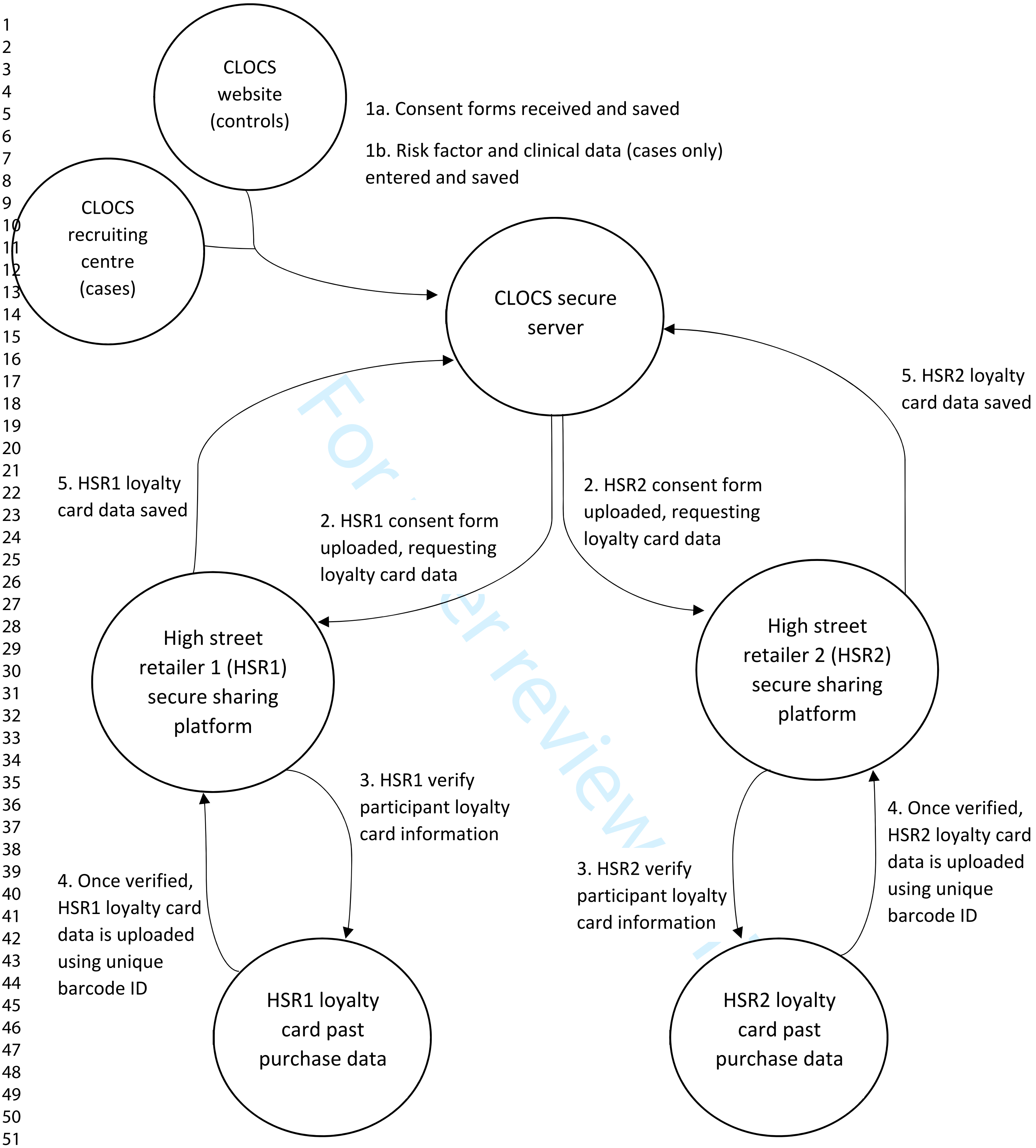
CLOCS website (controls)
CLOCS recruiting centre (cases)
1a. Consent forms received and saved
1b. Risk factor and clinical data (cases only) entered and saved
CLOCS secure server
5. HSR1 loyalty card data saved
2. HSR1 consent form uploaded, requesting loyalty card data
2. HSR2 consent form uploaded, requesting loyalty card data
5. HSR2 loyalty card data saved
High street retailer 1 (HSR1) secure sharing platform
High street retailer 2 (HSR2) secure sharing platform
3. HSR1 verify participant loyalty card information
4. Once verified, HSR1 loyalty card data is uploaded using unique barcode ID
3. HSR2 verify participant loyalty card information
4. Once verified, HSR2 loyalty card data is uploaded using unique barcode ID
HSR1 loyalty card past purchase data
HSR2 loyalty card past purchase data

Supplementary Data

Appendix A

Table 1: A list of example target items and behaviours to be analysed using loyalty card data.

**Purchases and behaviours to be explored:**	**Examples:**
Pain relief medications	Paracetamol, ibuprofen, aspirin, heat patch, etc.
Indigestion relief medications	Gaviscon, TUMS, Milk of Magnesia, omeprazole, etc.
Bloating and constipation relief medications	Imodium, Colpermin, Senokot, etc.
Change in diet	Sudden change to 'free from' foods such as gluten free or dairy free, or significant increase in purchasing high fibre foods, etc.

Appendix B

The following STROBE statement for case-control studies is the checklist CLOCS will use to report findings from the case-control study described in this protocol.

STROBE Statement—Checklist of items that should be included in reports of ***case-control studies***

	**Item No**	**Recommendation**
**Title and abstract**	1	(*a*) Indicate the study's design with a commonly used term in the title or the abstract
		(*b*) Provide in the abstract an informative and balanced summary of what was done and what was found
**Introduction**		
Background/rationale	2	Explain the scientific background and rationale for the investigation being reported
Objectives	3	State specific objectives, including any prespecified hypotheses
**Methods**		
Study design	4	Present key elements of study design early in the paper
Setting	5	Describe the setting, locations, and relevant dates, including periods of recruitment, exposure, follow-up, and data collection
Participants	6	(*a*) Give the eligibility criteria, and the sources and methods of case ascertainment and control selection. Give the rationale for the choice of cases and controls
		(*b*) For matched studies, give matching criteria and the number of controls per case
Variables	7	Clearly define all outcomes, exposures, predictors, potential confounders, and effect modifiers. Give diagnostic criteria, if applicable
Data sources/ measurement	8*	For each variable of interest, give sources of data and details of methods of assessment (measurement). Describe comparability of assessment methods if there is more than one group
Bias	9	Describe any efforts to address potential sources of bias
Study size	10	Explain how the study size was arrived at
Quantitative variables	11	Explain how quantitative variables were handled in the analyses. If applicable, describe which groupings were chosen and why
Statistical methods	12	(*a*) Describe all statistical methods, including those used to control for confounding
		(*b*) Describe any methods used to examine subgroups and interactions
		(*c*) Explain how missing data were addressed
		(*d*) If applicable, explain how matching of cases and controls was addressed
		(*e*) Describe any sensitivity analyses

Results

For peer review only

For peer review only - http://bmjopen.bmj.com/site/about/guidelines.xhtml

Participants	13*	(a) Report numbers of individuals at each stage of study—eg numbers potentially eligible, examined for eligibility, confirmed eligible, included in the study, completing follow-up, and analysed
		(b) Give reasons for non-participation at each stage
		(c) Consider use of a flow diagram
Descriptive data	14*	(a) Give characteristics of study participants (eg demographic, clinical, social) and information on exposures and potential confounders
		(b) Indicate number of participants with missing data for each variable of interest
Outcome data	15*	Report numbers in each exposure category, or summary measures of exposure
Main results	16	(*a*) Give unadjusted estimates and, if applicable, confounder-adjusted estimates and their precision (eg, 95% confidence interval). Make clear which confounders were adjusted for and why they were included
		(*b*) Report category boundaries when continuous variables were categorized
		(*c*) If relevant, consider translating estimates of relative risk into absolute risk for a meaningful time period

For peer review only

Other analyses	17	Report other analyses done—eg analyses of subgroups and interactions, and sensitivity analyses
**Discussion**		
Key results	18	Summarise key results with reference to study objectives
Limitations	19	Discuss limitations of the study, taking into account sources of potential bias or imprecision. Discuss both direction and magnitude of any potential bias
Interpretation	20	Give a cautious overall interpretation of results considering objectives, limitations, multiplicity of analyses, results from similar studies, and other relevant evidence
Generalisability	21	Discuss the generalisability (external validity) of the study results
**Other information**		
Funding	22	Give the source of funding and the role of the funders for the present study and, if applicable, for the original study on which the present article is based

*Give information separately for cases and controls.

Note: An Explanation and Elaboration article discusses each checklist item and gives methodological background and published examples of transparent reporting. The STROBE checklist is best used in conjunction with this article (freely available on the Web sites of PLoS Medicine at http://www.plosmedicine.org/, Annals of Internal Medicine at http://www.annals.org/, and Epidemiology at http://www.epidem.com/). Information on the STROBE Initiative is available at http://www.strobe-statement.org.

For peer review only

For peer review only - http://bmjopen.bmj.com/site/about/guidelines.xhtml
